# Intron retention as an excellent marker for diagnosing depression and for discovering new potential pathways for drug intervention

**DOI:** 10.3389/fpsyt.2024.1450708

**Published:** 2024-09-19

**Authors:** Norihiro Okada, Kenshiro Oshima, Akiko Maruko, Mariko Sekine, Naoki Ito, Akino Wakasugi, Eiko Mori, Hiroshi Odaguchi, Yoshinori Kobayashi

**Affiliations:** ^1^ School of Pharmacy, Kitasato University, Minato-ku, Tokyo, Japan; ^2^ Kitasato University Kitasato Institute Hospital, Minato-ku, Tokyo, Japan; ^3^ Oriental Medicine Research Center, School of Pharmacy, Kitasato University, Minato-ku, Tokyo, Japan

**Keywords:** depression, intron retention, marker, RNA-Seq, herbal medicine, treatment-resistant depression, innate immunity, cilium

## Abstract

**Background:**

Peripheral inflammation is often associated with depressive disorders, and immunological biomarkers of depression remain a focus of investigation.

**Methods:**

We performed RNA-seq analysis of RNA transcripts of human peripheral blood mononuclear cells from a case-control study including subjects with self-reported depression in the pre-symptomatic state of major depressive disorder and analyzed differentially expressed genes (DEGs) and the frequency of intron retention (IR) using rMATS.

**Results:**

Among the statistically significant DEGs identified, the 651 upregulated DEGs were particularly enriched in the term “bacterial infection and phagocytosis”, whereas the 820 downregulated DEGs were enriched in the terms “antigen presentation” and “T-cell proliferation and maturation”. We also analyzed 158 genes for which the IR was increased (IncIR) and 211 genes for which the IR was decreased (DecIR) in the depressed subjects. Although the Gene Ontology terms associated with IncIR and DecIR were very similar to those of the up- and downregulated genes, respectively, IR genes appeared to be particularly enriched in genes with sensor functions, with a preponderance of the term “ciliary assembly and function”. The observation that IR genes specifically interact with innate immunity genes suggests that immune-related genes, as well as cilia-related genes, may be excellent markers of depression. Re-analysis of previously published RNA-seq data from patients with MDD showed that common IR genes, particularly our predicted immune- and cilia-related genes, are commonly detected in populations with different levels of depression, providing validity for using IR to detect depression.

**Conclusion:**

Depression was found to be associated with activation of the innate immune response and relative inactivation of T-cell signaling. The DEGs we identified reflect physiological demands that are controlled at the transcriptional level, whereas the IR results reflect a more direct mechanism for monitoring protein homeostasis. Accordingly, an alteration in IR, namely IncIR or DecIR, is a stress response, and intron-retained transcripts are sensors of the physiological state of the cytoplasm. The results demonstrate the potential of relative IR as a biomarker for the immunological stratification of depressed patients and the utility of IR for the discovery of novel pathways involved in recovery from depression.

## Introduction

In 2017, it was estimated that more than 320 million people worldwide were affected by clinical depression (1). It is likely that this number—along with the number of people affected by other mental disorders—has since increased due to the ever-increasing stresses of daily life, particularly since the outbreak of COVID-19. In fact, depression has become the leading cause of disability worldwide. Major depressive disorder (MDD) is often accompanied by an anxiety disorder, and this combination is the leading cause of death by suicide (1). Currently, there is no reliable laboratory test or effective treatment strategy to diagnose or cure MDD. Another important issue in depression is the low remission rate, with only about half of patients achieving complete remission and the remission rate decreasing with each subsequent treatment. Therefore, to better understand the pathogenesis of depression and its etiology, there is an urgent need to identify biomarkers for monitoring treatment outcomes as well as genes that can be targeted for drug therapy (2–6).

There is increasing evidence that the incidence of peripheral inflammation correlates with that of depression (7–14). Several case-control studies of patients with MDD have reported elevated peripheral blood levels of inflammatory cytokines such as C-reactive protein, interleukin 6, and tumor necrosis factor (15–17). In these cases, where inflammation occurs first and depressive symptoms appear later, the view that inflammation contributes to depression is gaining ground. Furthermore, the prevalence of depression as a comorbidity is quite high in many inflammatory diseases that do not have a psychiatric comorbidity, such as rheumatoid arthritis (18, 19), suggesting a possible role for inflammation in depression. In his excellent book “THE INFLAMED MIND” (19), Ed Bullmore proposed that stress causes inflammation and that inflammation causes depression. Much of the current data seems to be consistent with his proposal (20).

Alternative pre-mRNA splicing is a mechanism by which multiple protein isoforms can be produced from a single gene transcript. One type of alternative splicing is called intron retention (IR), which was previously thought to simply reflect an error in pre-mRNA splicing. More recently, however, IR has been suggested to be a biologically meaningful phenomenon, as increases or decreases in intron abundance among specific transcripts have been associated with certain phenomena such as cell differentiation (21–24), aging (25), and oncogenesis (26). Using mouse models of aging such as Klotho mice (27) and SAMP8 mice (28), we have previously shown that the abundance of IRs increases in response to stress in the pre-symptomatic state and that when the state is restored by administration of a Japanese herbal medicine, the abundance of IRs is restored to that of the healthy state (27, 28).

Based on the above data, we hypothesize that IR is a modulator of gene expression through fine-tuning that occurs during RNA processing, although of course the primary regulation of gene expression occurs at the transcriptional level through the involvement of transcription factors. The stress condition, even before the obvious changes in transcription, requires fine-tuning of gene expression. The accelerator is expressed as a decrease in introns (called DecIR) and the brake as an increase in introns (called IncIR). The type of stress determines which genes require fine-tuning during stress. Not every gene can be regulated by intron retention, but of the 20,000 to 30,000 genes in the entire genome, 10 to 20% are likely to be selected to undergo IR such that their expression needs to be most tightly controlled during stress. As our previous studies and others have shown (25, 27), the loci of genes that undergo IR are relatively GC-rich and have short intron lengths. In other words, the set of genes that undergo IR during stress is genetically predetermined, and the total set of genes observed as IR genes can be considered to define the nature of that stress. Based on the above, genes affected by IR (hereafter referred to as IR genes) may play the role of sensors to detect perturbations in cellular homeostasis (29).

Therefore, we reasoned that an analysis of IR genes could facilitate the identification of stressors experienced by patients and the possibility that dysfunctional genes may underlie their depression. In this context, we explored the possibility that the incidence of IR could be used to investigate the etiology of depression.

## Materials and methods

### Ethics declarations, ethics approval and consent to participate

The research plan was reviewed and approved by the Research Ethics Committee of Kitasato Institute Hospital and assigned research number 21037. The study on which this research was based was an interventional study with the following approval numbers: No. 21039 and UMIN Study ID UMIN000045707. The Kitasato Institute Hospital Research Ethics Committee deliberates in accordance with the Ethical Guidelines for Medical and Health Research Involving Human Subjects in Japan. All participants provided written informed consent for the research procedures, including genetic analysis.

### Subjects

We recruited subjects with depressive symptoms who had agreed to participate in the “Study of Hangekobokuto (30) and the Intestinal Environment” conducted by the Kitasato University Oriental Medicine Research Center and who scored between 6 and 20 on the Brief Depressive Symptom Scale. After the benefits and risks of the study were explained to each subject, written informed consent was obtained. For subjects who consented, we applied the following eight exclusion criteria. 1) subjects who were already receiving medication for depression; 2) subjects who had taken herbal medicinal preparations within the previous 4 weeks; 3) subjects who had taken antibiotics within the previous 4 weeks; 4) subjects who were clearly in need of conventional medical treatment; 5) subjects who had been diagnosed with ulcerative colitis or Crohn’s disease; 6) subjects with clinically significant hepatic or renal impairment; 7) subjects who had participated in other clinical trials within the previous 12 weeks; and 8) subjects who were deemed by the investigators to be unsuitable for the study.

Each subject took a single daily dose of the Japanese herbal medicine Hangekobokuto (HKT) at home. The BDI™-II Beck Depression Questionnaire (BDI-II) was administered at the time of initial screening and at hospital visits 2 months after the last dose of HKT, and blood was drawn using a BD Vacutainer CPTTM Blood Collection Tube (Nippon Becton Dickinson, Japan). Subjects were classified according to their BDI-II score, with six subjects scoring less than 16 and being considered as controls (CON) and eight subjects with depression symptoms (before medical treatment, BMT, or after medical treatment, AMT) scoring 17 or higher.

### Japanese herbal medicine

Japanese herbal medicines originated in ancient China and are widely used in Japan to treat a variety of conditions (31, 32). HKT (30) is one such formulation and is taken for symptoms of anxiety, stagnant gas in the stomach, and poor digestive function. In this study, HKT was used as a decoction in the following amounts, based on the formula from the Kitasato University Oriental Medicine Research Center: Hange (Pinelliae Tuber) 6.0 g; Bukuryo (Hoelen) 5.0 g; Koboku (Magnoliae Cortex) 3.0 g, Shisoyo (Perillae Herba) 2.0 g; Syokyo (Zingiberis Rhizoma) 0.5 g.

### PBMCs preparation, RNA extraction, RNA library preparation and RNA-sequencing

Blood samples collected in BD Vacutainer CPTTM Blood Collection Tubes are centrifuged within 2 hours to separate the peripheral blood mononuclear cell (PBMC) layer. After centrifugation, PBMC samples can be stored and transported at -80°C (long term storage box).

RNA extraction was performed on individual PBMC samples using the Pure Link RNA Mini Kit (Invitrogen, MA, USA). Briefly, 500 μL lysis buffer and 750 μL TRIzol (Thermo Fisher Scientific) were added to 0.03 g PBMC, and the cells were homogenized. After incubation for 15 minutes at room temperature and centrifugation at 12,000 × g for 10 minutes, the supernatant was ethanol precipitated and collected in a column cartridge. During this RNA purification process, on-column digestion of DNA was performed using DNase. The quality of the RNA was checked using a Qubit (Thermo Fisher Scientific) and a TapeStation (Agilent Technologies, CA, USA). The RNA integrity numbers (RIN) of these RNA samples ranged from 6.4 to 9.6. Library construction and paired-end sequencing (150 base pairs × 2) using the NovaSeq 6000 platform (Illumina) were outsourced to Azenta Life Sciences, Tokyo, Japan.

RNA sequencing yielded 109 ~ 148 million (× 2, paired-ends) raw reads per sample. Sequencing data were cleaned by removing Illumina adapter sequences using cutadapt v.1.16, followed by the fastx toolkit software package v.0.0.14 (http://hannonlab.cshl.edu/). The fastx_clipper software from fastx_toolkit/was used to remove poly(A) sequences. To remove low quality bases and sequences, the sequences were trimmed using the fastq_quality_trimmer software (parameters: -t 20 -l 30 -Q 33) and the fastq_quality_filter software (parameters: -q 20 -p 80 -Q 33) was used to filter the sequences. During the above process, reads with one of the missing pairs were removed using Trimomatic v.0.38. Next, reads containing human rRNA, tRNA, globin related gene sequences (HBA1, HBA2, HBB, HBD, HBM, HBG1, HBG2, HBE1, HBQ1 and HBZ), phiX sequences as control sequences from Illumina were removed using Bowtie 2 v.2.3.4.1. A second unpaired read removal was then performed using bam2fastq. After these filters, 90 million (× 2, paired-ends) reads per sample were mapped to the human genome sequence NCBI GRCh38 using HISAT2 version 2.1.0. Multiple mapping reads were removed using samtools (parameter: samtools view -q 4) and unique mapping reads were counted by gene annotation (NCBI Homo sapiens Annotation release 106) using FeatureCounts v.2.0.0. Counts were normalized by the trimmed mean of M values (TMM) method using the EdgeR library in R v.4.2.0 and used for expression analysis (33–36). A schematic representation of RNA-seq data processing is shown in **Supplementary Figure 1A**.

### Analysis of differentially expressed genes

Using the edgeR package in R, significantly differentially expressed genes (DEGs) in patients with depression were detected by performing the likelihood ratio test. The results showed that 922 downregulated and 641 upregulated genes were significantly differentially expressed between the six CONs and eight BMTs (p<0.05 and fold-change > 1.2 or FDR<0.1 and fold change >1.2). DEGs were used for GO (Gene Ontology) and KEGG (Kyoto Encyclopedia of Genes and Genomes) pathway-enrichment analysis using the DAVID website. Similarly, the same likelihood ratio test values were calculated under the same conditions between BMT and AMT to investigate the effect of HKT administration.

### Detection of IR

IR-containing genes were analyzed to determine their possible role in stress sensing as proposed previously (27–29). rMATS v.4.1. was used to assess the differential IR landscape embedded in the RNA-seq data. For our analysis, the parameters for the rMATS program were as follows: [–cstat 0.05 -t paired –readLength 150 –variable-read-length]. A cut-off of p<0.05 or FDR<0.1 in the likelihood ratio test and an absolute difference of the IR ratio > 0.05 (both used to establish statistical significance in the rMATS program) were used to call differential IR events. Similarly, the same likelihood ratio test values were calculated under the same conditions between BMT and AMT to study the effect of HKT administration.

### Interactome analysis

A protein-protein interaction network was generated using Cytoscape ver. 3.9.1 with StringApp version 1.7.1. The network type “full STRING network” was selected for plotting graphs, and a confidence-score cut-off value of 0.7 was used (default values were used for all other parameters). Data presented in the present study was established using proteins encoded by IR genes and DEGs, and protein-protein interactions were analyzed among members of each functional gene group, cilia-related genes (proteins), psychiatric disorders–relevant genes (proteins), and adaptive and innate immunity–related genes (proteins).

## Results

### RNA-seq and analysis of DEGs

**Figure 1:**
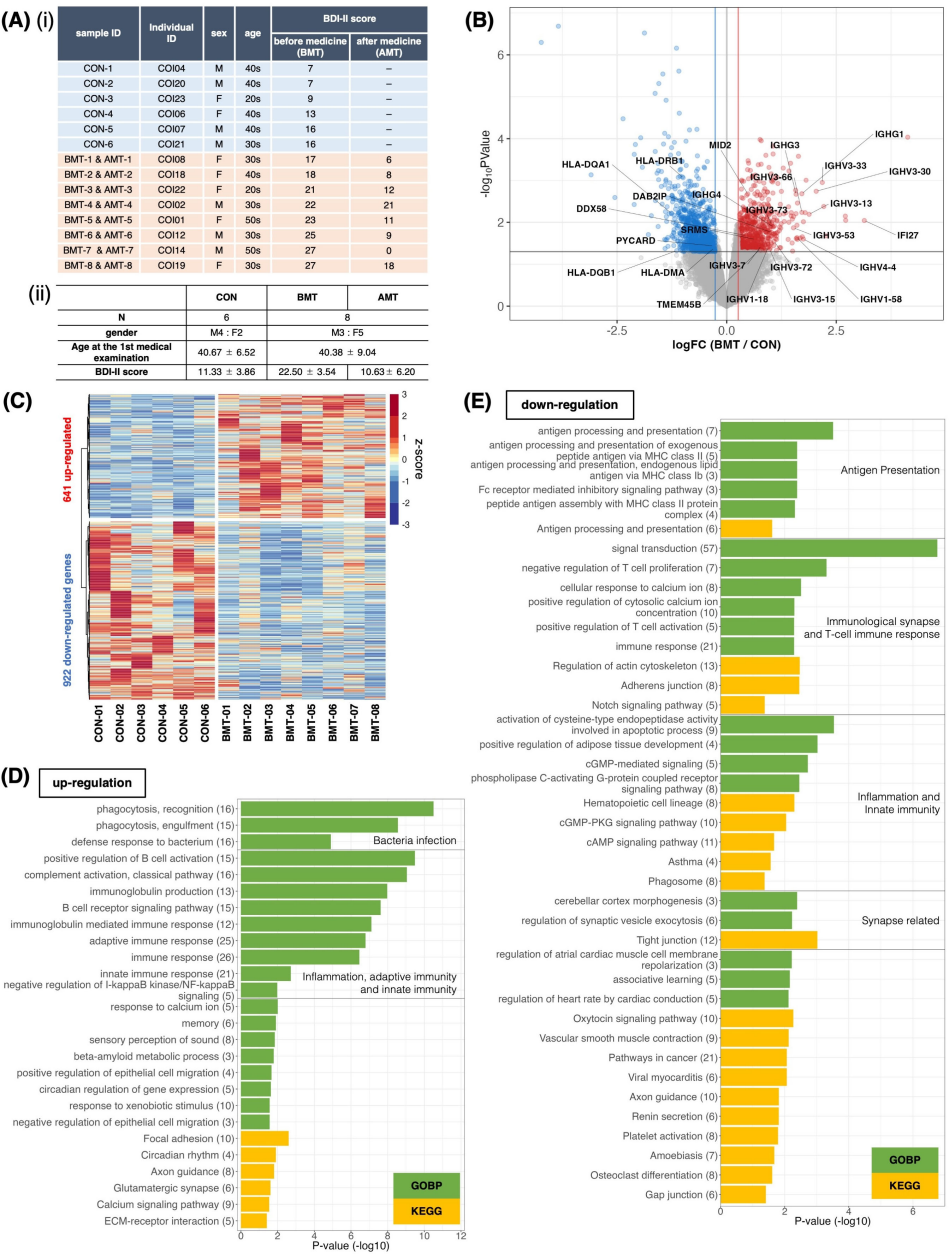
Comparison of RNA expression between depressed subjects and less depressed controls. **(A)** (і) Subject information. Classification was based on the BDI-II score at first examination. Subjects with BDI-II score ≤ 16 were categorized as less depressed (controls, CON), and those with BDI-II score > 16 were categorized as depressed. BMT, depressed subjects before medical treatment; AMT, depressed subjects after 2 months of taking HKT. **(ii)** Mean and standard deviation of sex, age, and BDI-II score for each group. **(B)** Volcano plot of RNA expression between the BMT and CON groups. The horizontal axis shows the log_2_ fold-change of BMT/CON, and the vertical axis shows –log_10_ *P*-values. Red dots denote significantly upregulated genes (FC (fold change) > 1.2 and p<0.05), blue dots denote significantly downregulated genes (FC < 1/1.2 and p<0.05), and grey dots indicate no significant difference in expression (likelihood ratio test). Gene symbols for T cell–associated genes are indicated. **(C)** Heatmap of significantly differentially expressed genes between BMT and CON subjects. **(D, E)** Enrichment analysis of biological processes among Gene Ontology and KEGG pathway terms for the 641 upregulated **(D)** or 922 downregulated **(E)** genes in the BMT group. The horizontal axis shows –log_10_ *P*-values. Green bars indicate Gene Ontology biological process terms, and yellow bars indicate KEGG pathway terms.

The study included a group of eight subjects with depression whose BDI-II scores ranged from 17 to 27, reflecting moderate depression. These subjects were referred to as the BMT group. Six other subjects with relatively mild depression had scores ranging from 7 to 16, and these subjects served as controls (CON group). [see **Figure 1A**(i)(ii)]. As we are all on the spectrum, we wanted to gain molecular insight into the transition from mild to moderate depression. All subjects were also screened to ensure that they had not taken any medication in the one month prior to the study or been hospitalized in the 3 months prior to the study (see Materials and Methods for details). Accordingly, all SUBJECTS analyzed here have never been patients with MDD and have no medical history. The purpose of this study is to determine if it is possible to detect changes in intron retention at such an early stage of depression, which is not pathological and can occur in anyone.

**Figure 2:**
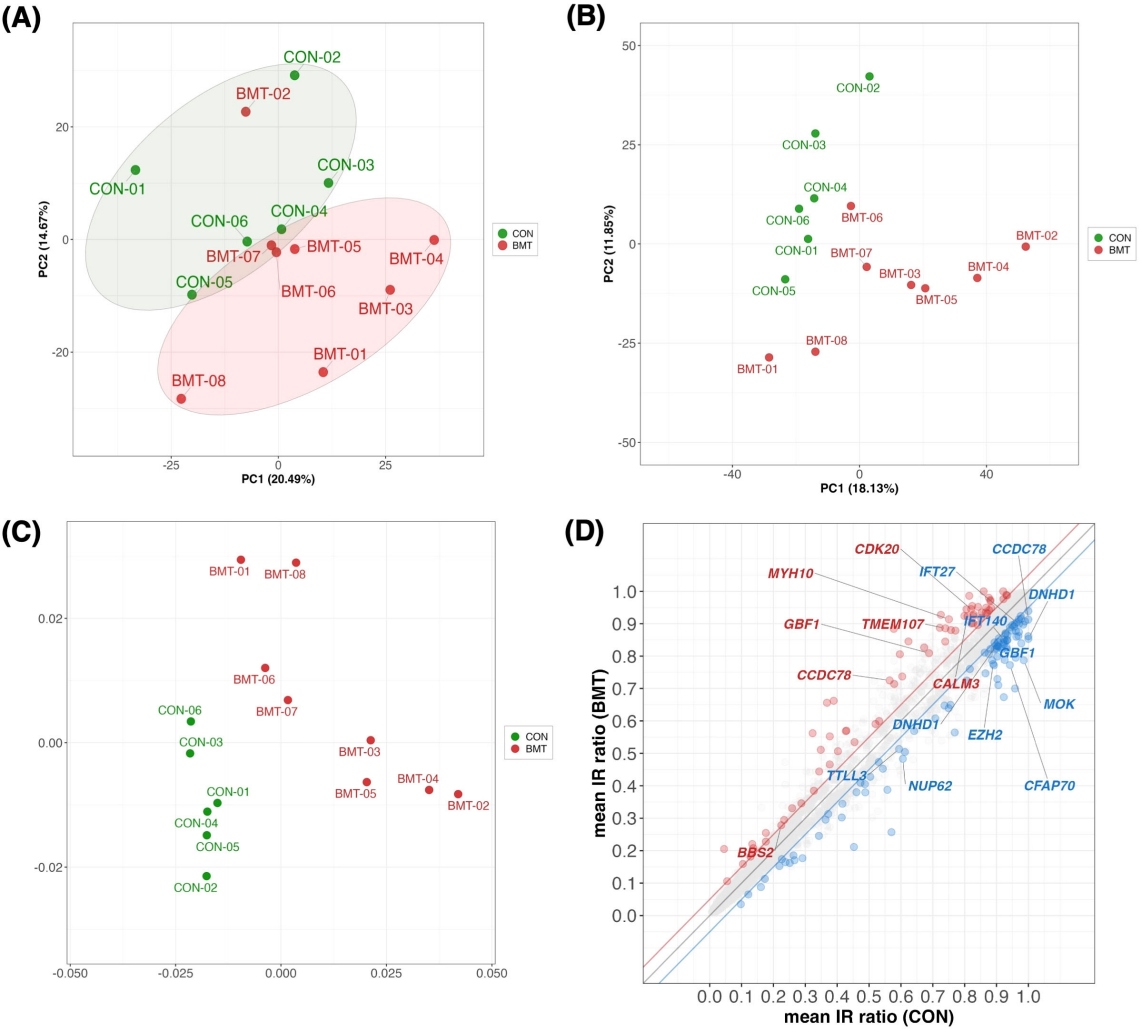
Multivariate statistics analysis between CON and BMT. **(A)** Principal component analysis (PCA) plot with CON and BMT using 1,658 of the 2,738 genes in the “GO:0006955 immune response” with a TMM normalized value > 1 (RNA expression). **(B)** The IR ratios at 10,286 loci with defined IRs from the annotation file were calculated by rMATS for 3,140 loci with a coverage of > 10 reads. PCA plots with color coding in CON and BMT (using PC1 and PC2). **(C)** MDS plots with color coding in CON and BMT. **(D)** Scatterplot showing mean CON and mean BMT for IR ratios at 3,140 loci. Red indicates IncIR and blue indicates DecIR. Gene names indicate cilium-related genes with significant differences in IncIR and DecIR. (Thresholds are FDR < 0.1, absolute IR difference > 0.05 and IR junction with a coverage of >10 reads).

PBMC and RNA were isolated from each sample and used for RNA-seq. DEG analysis identified 651 upregulated and 820 downregulated genes for the BMT group compared to the CON group at p<0.05 (**Figures 1B, C**, **Supplementary Table 1**). With FDR<0.1, 20 upregulated and 43 downregulated genes were identified (**Supplementary Figure 1**, **Supplementary Table 1**). GO enrichment analysis of upregulated genes at p<0.05 (**Figure 1D**, **Supplementary Figure 2** for details) revealed enrichment for innate immunity–related terms such as infection, phagocytosis, inflammation and adaptive immunity–related terms; the analysis of downregulated genes (**Figure 1E**, **Supplementary Figure 3** for details) revealed enrichment for adaptive immunity–related terms such as antigen presentation, T-cell activation, and synapse-related terms. **Figure 1B** shows all downregulated genes, including those involved in T-cell activation (5 genes in **Figure 1E**), and all upregulated genes, including those involved in the innate immune response (21 genes in **Figure 1D**). It is interesting to note that the latter includes a large number of immunoglobulin heavy chains (37, 38), suggesting that many of the subjects had an inflammatory phenotype (37). In addition, principal component analysis (PCA) with CON and BMT using 1,658 genes categorized as GO immune response showed that individuals with CON were clearly separated from those with BMT, making it a rational to separate subjects into these two groups (**Figure 2A**).

In short, the study analyzed two groups of subjects with depression: a BMT group with moderate depression (BDI-II scores 17-27) and a CON group with mild depression (BDI-II scores 7-16). Enrichment analysis revealed that upregulated genes were associated with innate immunity and downregulated genes were associated with adaptive immunity. PCA confirmed distinct molecular profiles between CON and BMT even at a very early stage.

### Identification of IncIR and DecIR genes in depressed subjects

**Figure 3:**
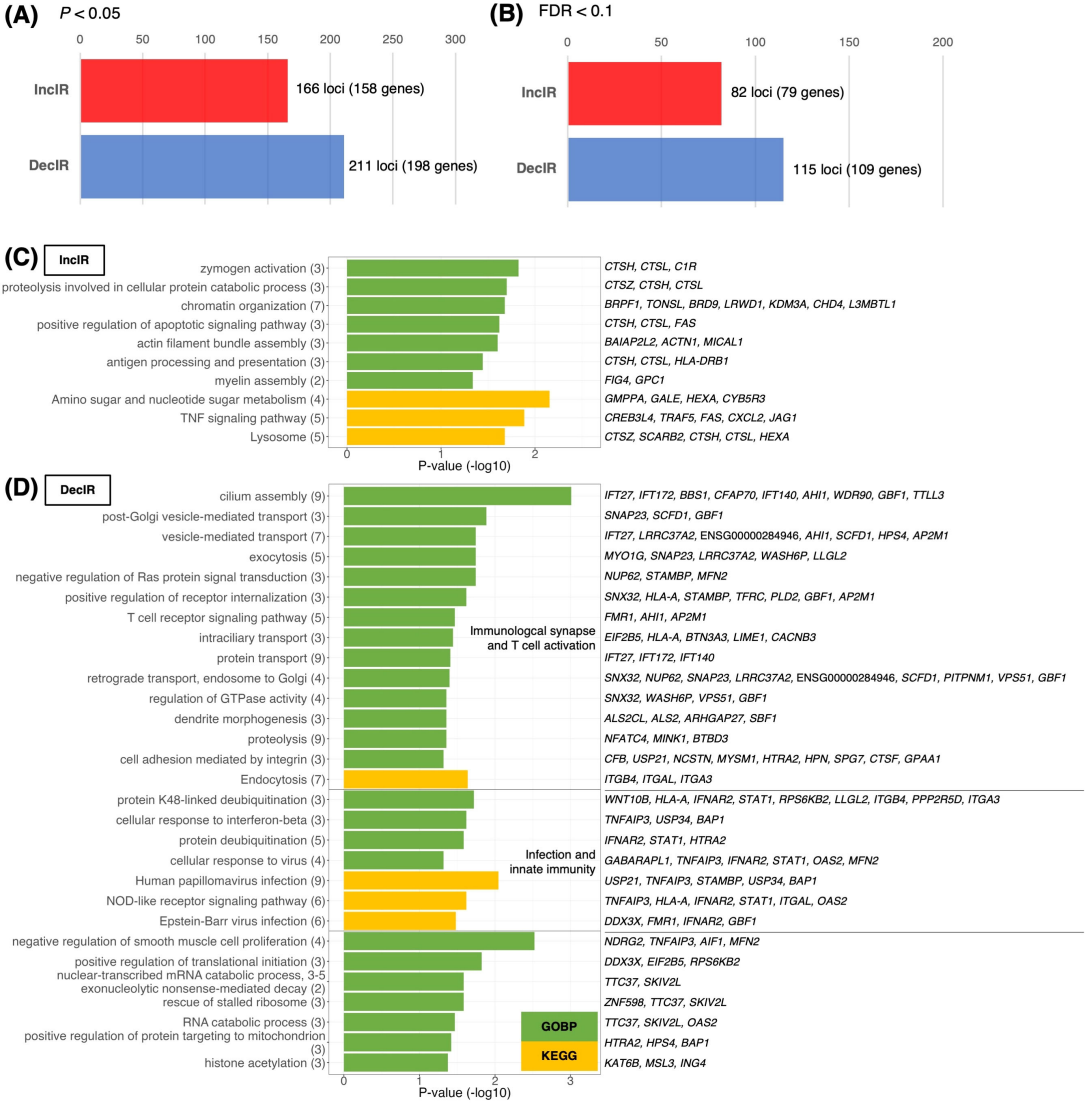
Identification and characterization of IR genes. **(A, B)** Number of IR genes with significantly increased IR (IncIR) or decreased IR (DecIR) for BMT versus CON using rMATS software v.4.1.1. The statistical significance of differences between values was based on a P-value < 0.05 **(A)** or FDR < 0.1 **(B)** and a difference in intron ratio of > 0.05. **(C, D)** Enrichment analysis of biological processes among Gene Ontology and KEGG pathway terms using 158 IncIR genes **(D)** and 198 DecIR genes **(D)** in the BMT group (*P* < 0.05). Gene symbols corresponding to the terms are shown on the right. The horizontal axis shows –log_10_ *P*-values. Green bars indicate Gene Ontology biological process terms, and yellow bars indicate KEGG pathway terms.

Since IR is a stress response and genes susceptible to IR are a physiological sensor [(27, 28); see Introduction and later discussion], we characterized genes for which IR was increased in depressed subjects (IncIR) and genes for which IR was decreased (DecIR), reasoning that such an analysis would indicate the type of stress to which the subjects had been exposed. A total of 158 IncIR and 198 DecIR genes were identified between CON and BMT at p<0.05 (**Figure 3A**, **Supplementary Table 2**). With FDR<0.1, 79 genes with significantly increased IR and 109 with significantly decreased IR were characterized (**Figure 3B**, **Supplementary Table 2**). Their characteristics were first investigated based on published information. As expected, many sensor or regulatory genes were represented among the protein-coding IR genes (**Table 1**; 45 genes, of which 27 genes in bold were detected at FDR<0.1). This is only half of the genes identified as sensors, regulators and modulators among the IR genes in this analysis, in which genes controlling inflammation, innate immunity and adaptive immunity were identified. GO enrichment analysis (**Figures 3C, D**) of IncIR revealed enrichment of terms related to the TNF signaling pathway and several terms related to the innate immune response. Among the DecIR genes, there was enrichment of terms related to T-cell signaling and other adaptive immune responses as well as inflammation and innate immune processes. In short, both innate and adaptive immunity were highlighted in the IR analysis, as in the DEG analysis, suggesting that the IR genes mirror the DEG genes. The important difference between the IR genes and the DEG genes is that immunoglobulin genes were included in the DEG list - in fact, almost half of the upregulated genes in our RNA-seq analysis were immunoglobulin genes (see **Supplementary Figure 2**), but this was not true for the IR genes (see Discussion). The reason why immunoglobulin genes were not among the IR genes will be discussed later. Interestingly, the highest enrichment score among the DecIR genes was for cilium assembly, suggesting that cilia are involved in sensing depression-induced stress (see Discussion). In addition, we performed PCA (**Figure 2B**) and MDS (**Figure 2C**) analyses by using the IR data of 3,140 loci between CON and BMT obtained by rMats, and showed that these two groups of CON and BMT were clearly separated by both methods, providing another rationale for the availability of IR to assess the depressive state of the subjects.

**Table 1:** Sensor and regulatory genes that were identified among the protein-coding IR genes.

Category	Gene *	Function	Ref.
Sensor	***ADGRB2***	Metabotropic mechanosensor	(100)
	***AIF1***	Innate response sensor	(101)
	*CD163*	Macrophage innate immune sensor	(102)
	*DDX5*	Interferon antiviral sensor	(103)
	***DDX3X***	Interferon antiviral sensor	(103)
	*ERLIN1*	Innate immune sensor	(104)
	***GBF1***	ER_sensor	(105)
	*HTRA2*	Mitochondria stress sensor	(106)
	*LRSAM1*	Bacterial sensor	(107)
	*MAP3K12 (DLK)*	axon-damage sensor	(108)
	***MOK (RAGE)***	Heme sensor	(109)
	*NDRG2*	Inflammation sensor	(110)
	***NFATC4***	Nerve sensor	(111)
	***OAS2***	Viral sensor	(42)
	*PQBP1*	HIV innate response sensor	(112)
	*SARM1*	Metabolic sensor	(113)
	***SLC9A5 (NHE5)***	PH sensor	(114)
	***SLC16A11***	Glucose lipid sensor	(115)
	***ZNF598***	Collided_ribosome sensor	(116)
Regulator	***ADCY4***	Controls caspase-11 inflammasome activation	(117)
	***BRD9***	Regulates interferon-stimulated genes	(96)
	*BTBD3*	Controls dendrite orientation	(118)
	***BTN3A3***	Regulates ERK1/2 phosphorylation	(119)
	***CACNB3***	Regulates ATP-dependent migration of dendritic cells	(120)
	***CFB***	Regulates cellular senescence	(121)
	*CLK4*	Regulates DNA damage induced NF-kB	(122)
	***HAGHL***	Regulates human colorectal cancer progression	(123)
	*ITGAL*	Regulates glioma growth	(124)
	***MAT2B***	Regulates EGFR signaling pathway	(125)
	***METTL17***	Regulates mitochondrial ribosomal RNA modifications	(126)
	***MICAL1***	Regulates actin microfilaments	(127)
	***MSH5***	Regulates Ig class switch recombination	(128)
	*MYO1G*	Regulates exocytosis, and endocytosis in B lymphocytes	(129)
	*MYSM1*	Regulates hematopoietic stem cell maintenance	(130)
	*NAPEPLD*	Regulates liver lipid metabolism	(82)
	***PGM3***	Regulates beta-catenin activity	(131)
	***PLD2***	Regulates phagocyte cell migration	(132)
	***PTPN18***	Regulates the c-MYC-CDK4 axis	(133)
	*ROBO3*	Modulates prognosis via AXL-associated inflammatory network	(92)
	***SCFD1***	Regulates SNARE complex formation	(134)
	***SNAP23***	Regulates phagocytosis	(135)
	*STARD9*	Regulates Spindle Pole Assembly	(136)
	***STAT1***	Regulates transcription in the interferon JAK-STAT pathway	(39)
	***TRA2A***	Regulates EZH2/beta-catenin pathway	(137)
	*UBE2T*	Promotes autophagy	(138)

*Genes detected under the condition of FDR<0.1 are shown in bold. Others were detected at p<0.05.

In short, the analysis identified IR genes as sensing role in stress in depressed subjects and involvement of these genes associated with innate and adaptive immunity in modulating gene expression as IR. The study highlights that the IR genes mirror the DEG genes in function, but their essential differences exist. PCA and MDS analyses confirmed that the IR data effectively discriminated between control and depressed states.

### IR genes interact in a statistically significant manner with genes related to the innate immune response; potential makers for depression

**Figure 4:**
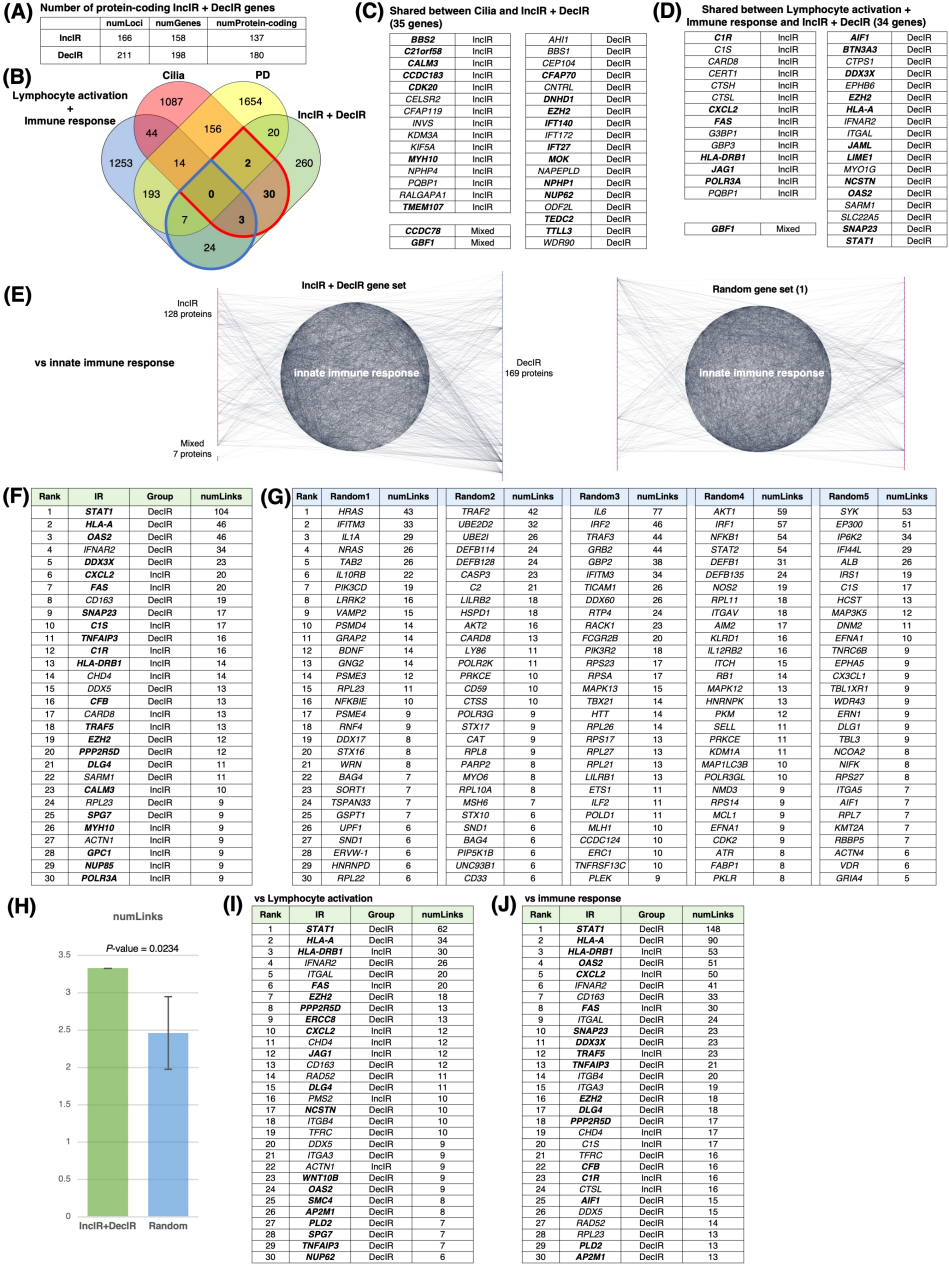
IR loci interact preferentially with genes involved in innate immunity. **(A)** Number of IR genes and IR protein-coding genes. (see **Figure 4A**) **(B)** Venn diagram between IR genes, cilia-related genes (GO: 0060271 cilium assembly + SCGSv2 ciliary genes (72) + CiliaCarta (160)), adaptive and innate immunity–related genes (GO: 0046649 lymphocyte activation and GO: 0006955 immune response), and psychiatric disorders–relevant (PD) genes. The PD gene set was constructed by merging genes from the following databases or previous studies: SFARI (autism-related gene database, https://www.sfari.org/resource/sfari-gene/); PsyGeNET (mental disorder-related gene database, https://www.sfari.org/resource/sfari-gene/); PD genes http://www.psygenet.org/web/PsyGeNET/menu/home); PD genes (73); major depression risk genes (74). **(C)** Gene symbols shared between IR and cilia-related genes, corresponding to the area outlined in red in **(B)** Groups were classified as IncIR, DecIR or Mixed (‘Mixed’ indicates a gene for which one intron was classified as IncIR and another classified as DecIR). **(D)** Gene symbols shared between IR and immunity–related genes, corresponding to the area outlined by blue in **(B, E)** Significant protein-protein interactions (PPI) between proteins encoded by IR genes and those encoded by innate immune response–related genes. The interaction score was calculated using the full STRING network confidence score 0.7 from the STRING database. (Left) Network of PPIs between proteins encoded by IR genes and innate immune response genes (GO: 0045087). Innate immune-response proteins were placed in the central circle and IR proteins (red: IncIR, blue: DecIR, green: Mixed) on either side. (Right) Instead of IR proteins, equal numbers of randomly selected gene sets were placed on both sides. Ranking table shows the top 30 proteins with the highest number of interactions (number of links) with the IR genes in **(F)** and with 5 randomly selected protein sets in **(G)**. **(H)** Comparison of the average number of interactions (number of links) with innate immune response proteins among the IR genes and 5 random protein sets. **(I)** Ranking table showing the top 30 proteins with the greatest number of interactions (links) to lymphocyte-activation proteins (GO: 0046649) among the IR proteins. **(J)** Ranking table showing the top 30 proteins with the greatest number of interactions (links) to immune-response proteins (GO: 0006955) among the IR proteins. Genes in bold in the tables (**C**, **D**, **F**, **I, J**) indicate those of IncIR or DecIR with FDR < 0.1.

To characterize the IR genes in more detail, we first determined the possible overlap of IR genes with immune-related genes, cilia genes, and psychiatric disease–related genes (designated PD; **Figures 4A, B**). The 317 IR genes (**Supplementary Table 2**) included 35 cilia genes (**Figure 4C**) and 34 immune-related genes (**Figure 4D**), with IR genes detected under the condition of FDR<0.1 shown in bold. The scatterplot in **Figure 2D** also showed cilium-related genes with significant differences in IncIR and DecIR.

Since many of the IR genes listed in **Table 1** are involved in innate immunity, including viral and bacterial infection, we next examined whether IR genes could specifically interact with genes involved in the innate immune response. Indeed, IR genes were found to specifically interact with genes involved in innate immunity compared to the same number of randomly selected genes (statistically significant, P < 0.0234; **Figures 4E, H**). **Figure 4F** shows the ranking of the IR genes in interactions with genes involved in innate immunity with FDR<0.1 shown in bold. The ranking was contrasted with those randomly selected as controls (**Figure 4G**). Among the IR genes, *STAT1* [signal transducer and activation of transcription gene 1; **Table 1** (39)] had the largest number of interactions with innate immunity genes (the number of links: 104). Notably, *STAT1* is involved in the JAK-STAT pathway (39), which contributes to both innate and adaptive immunity including inflammation (see Discussion). Using the same interaction assay system, interactions between IR genes and genes involved in leukocyte activation (adaptive immunity; **Figure 4I**) or the immune response (**Figure 4J**) were also examined, and it was found that *STAT1* ranked highest in each comparison (**Figures 4I, J**). It is interesting to note that IR genes that ranked higher in their interaction, represented by the number of links, such as *STAT1* (39)*, HLA-A* (40), *HLA-DRB1* (41), *OAS2* (42) and *CXCL2* (43), were selected under the condition of FDR<0.1 in the rMATs analysis between CON and BMT (shown in bold).

In short, out of 317 IR genes, 35 were related to cilia and 34 to immunity. IR genes interacted specifically with immune response genes, with *STAT1* showing the most interactions. The genes characterized in this section may be potential markers for depression.

### A protein-protein interaction network using genes for IR and DEG represents depression at the molecular level

**Figure 5:**
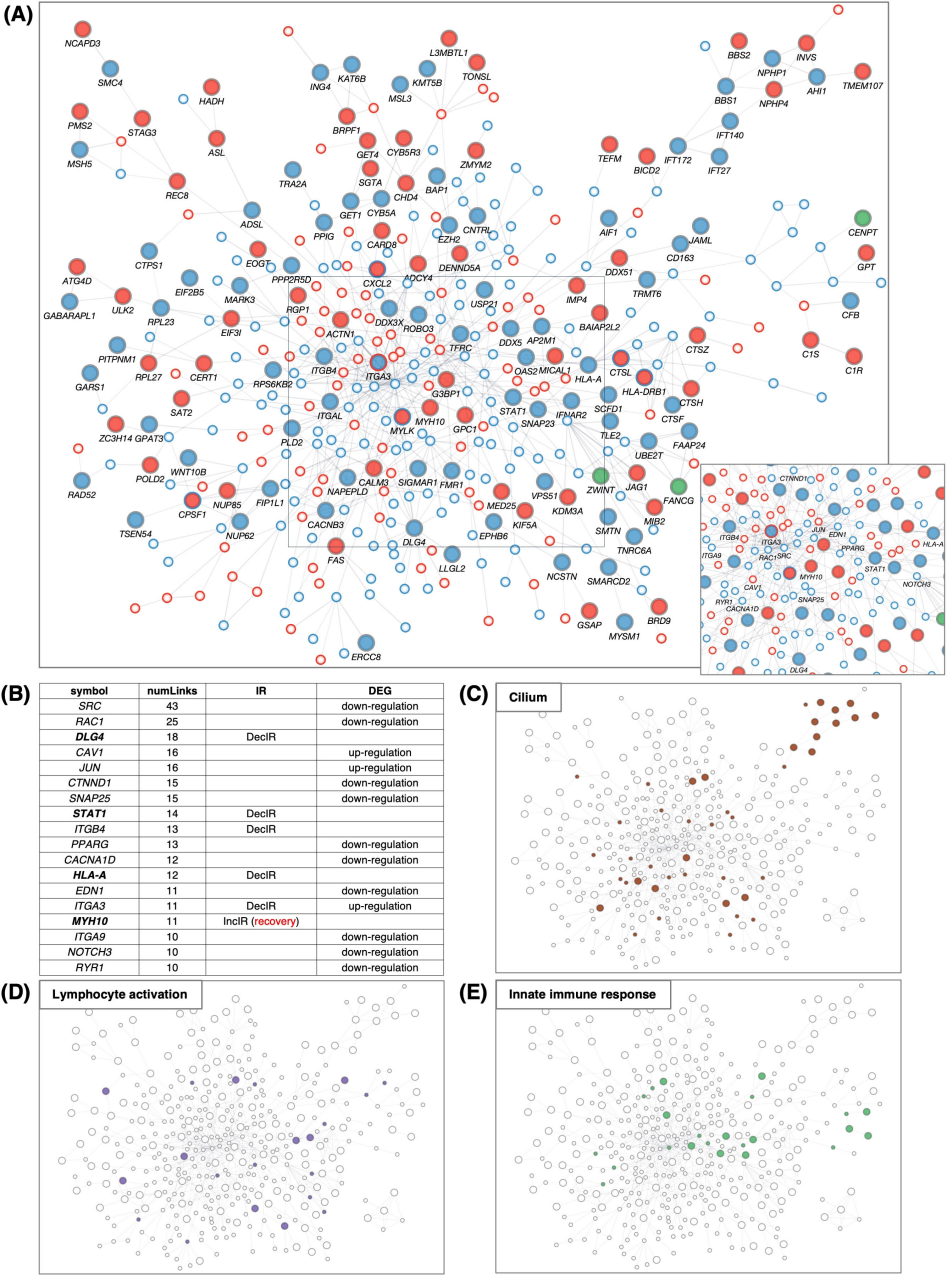
PPI network of IR and DEG proteins. **(A)** Main network showing PPIs using all IR and DEG proteins between BMT and CON. The interaction score was calculated using the full STRING network confidence score 0.7 from the STRING database. IncIR: red circles, DecIR: blue circles, Mixed: green circles. Upregulation: small circle with red border, downregulation: small circle with blue border. Only the largest networks are shown, as singletons and smaller networks were excluded. IR protein names are shown. Inset, bottom-right: (A square bottom right) Proteins with ≥10 interactions (number of links) are marked with symbols. **(B)** Ranking table of protein names with 10 or more interactions. Genes in bold in this table indicate those of IncIR or DecIR detected at FDR < 0.1. **(C–E)** Proteins corresponding to cilia (brown, GO: 0060271 cilium assembly) in **(C)**, lymphocyte activation (purple, GO: 0046649 lymphocyte activation) in **(D)**, and innate immunity (light green, GO: 0045087 innate immune response) in **(E)** are colored as in the network in panel **(A)**.

**Figure 6:**
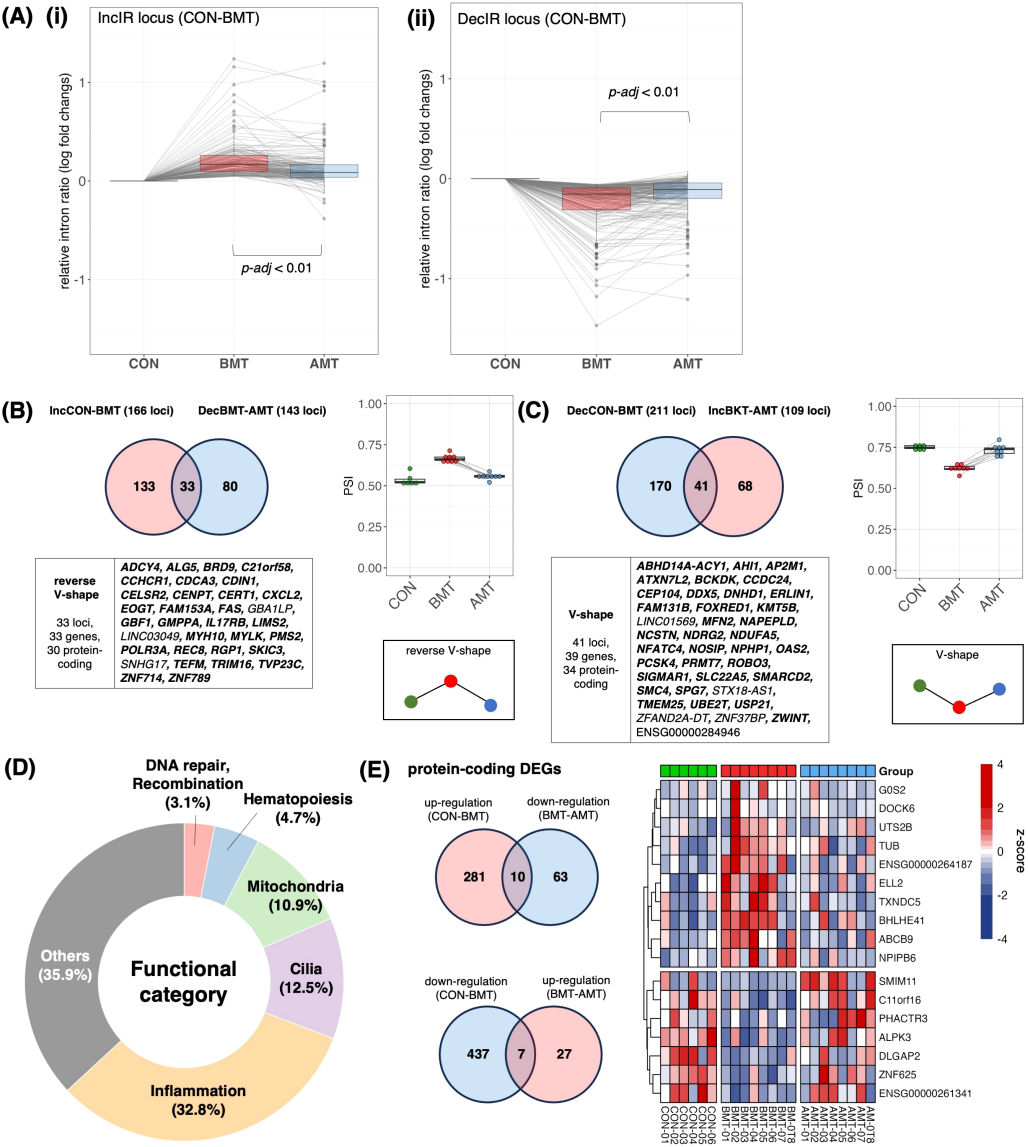
Recovery of IR by administration of HKT. **(A) (i)** Of the 166 loci that were IncIR in BMT compared with CON, the change in relative intron ratio in AMT was analyzed at 158 ​​loci excluding loci with low coverage in AMT. One-way ANOVA was used for testing, and Tukey’s test was used for multiple testing. **(ii)** Of the 211 loci that were DecIR in BKT compared with CON, the change in relative intron ratio in AMT was analyzed at 207 loci, excluding loci with low coverage in AMT. **(B)** (top, left) Venn diagram of intronic loci that were significantly increased (IncIR between CON and BMT) and those that were decreased (DecIR between BMT and AMT) by drug treatment. (bottom, left) Gene symbols with recovered loci are shown (Protein coding genes are shown in bold.). (Right) Box plot showing average intron ratios at recovered loci. **(C)** (Top, left) Venn diagram of intronic loci that were significantly decreased (DecIR between CON and BMT) and those that were increased (IncIR between BMT and AMT) by drug treatment. (Bottom, left) Gene symbols with recovered loci are shown (Protein coding genes are shown in bold.). (Right) Box plot showing average intron ratios at recovered loci. **(D)** Table of functional categorization from the literature of the recovered IR genes. **(E)** (top, left) Venn diagram of RNA expression that were significantly reverse V-shape recovery. (bottom, left) Venn diagram of RNA expression that were significantly V-shape recovery. (right) Heatmap showing z-score of protein-coding RNA expression of recovery genes.

Using all the protein-coding genes of the DEGs (285 upregulated + 433 downregulated) and IR genes (129 IncIR + 172 DecIR + 8 common to both) at p<0.05, we generated a large network (**Figure 5A**), termed IR-DEG interactome. Within the large interactome, many hub genes were connected to other genes (**Supplementary Table 4**). The largest hub was centered around *SRC* (44), one of the DEGs, which was connected to 43 genes (**Figure 5B**). Among the IR genes, the largest hub was *DLG4* (45), which is involved in synaptic function. The second was *STAT1*. The third and fifth were integrin genes (46, 47), and the fourth was *HLA-A* (40), which is involved in antigen presentation. The sixth was *MYH10* (48), myosin heavy chain, which has 11 links, one of which is linked to myosin light chain kinase (MYLK) (49, 50); importantly, the IR of the *MYH10* transcript was restored in subjects after administration of HKT (see Discussion). Genes related to the cilium (**Figure 5C**), lymphocyte activation (**Figure 5D**) and innate immune response (**Figure 5E**) were mapped on the IR-DEG interactome, providing a means to verify the identity of each gene after annotation of recovery genes (**Figure 6**).

### Recovery of IR genes by administration of HKT to subjects

After 2 months of HKT administration, PBMCs were isolated from the subjects’ blood samples, RNA-seq was performed and IR genes were identified. First, we confirmed an intron increase of 166 loci [**Figure 6A**(i)] and an intron decrease of 211 loci [**Figure 6A**(ii)] of BMT, those of which were identified by rMats, compared to that of CON. We then examined the change in the relative intron ratio of AMT to BMT after 2 months of HKT administration. In both cases, IncIR and DecIR, most IRs showed the recovery trend with AMT, suggesting the efficacy of HKT in the depressed subjects. We then characterized two types of statistically significant IR recoveries (p<0.05) for which IR was restored after HKT administration, namely reverse V-shape recovery in **Figure 6B**, which included 30 protein-coding genes, and V-shape recovery in **Figure 6C**, which included 34 protein-coding genes. Two types of statistically significant IR recoveries at FDR<0.1 were also shown in **Supplementary Figure 4**. Since only 17 genes (7 for the V-shape recovery and 10 for the reverse V-shape recovery) were recovered among those identified as DEGs (p<0.05; **Figure 6E**), the fact that IR was recovered in four times as many IR genes as DEGs after HKT treatment suggested that IR is more tightly linked to the efficacy of a drug and is superior to DEGs as a marker for evaluating its efficacy.

Characterization of the 64 protein-coding genes for which IR was restored after HKT treatment (**Figure 6D**) showed that inflammation-related genes were most affected (21 genes; 37.5%), with 8 cilia-related genes accounting for 12.5% and 7 mitochondria-related genes accounting for 10.9%. The anti-inflammatory effect shown here is consistent with the reported efficacy of many Japanese herbal medicines (31), including HKT (30). Interestingly, while HKT has been reported to have anti-inflammatory effects by restoring the activity of inducible nitric oxide synthase, the IR of the mRNA of the encoding gene, *NOSIP* (51), which modulates cellular NO levels, was consistently restored by HKT treatment (**Figure 6C**, **Table 2**). The identification of three hematopoiesis genes (**Figure 6D**, **Table 2**) may also indicate that inflammation and hematopoiesis are linked in a compensatory manner, as inflammation consumes a large number of macrophages that should be relenished. Oxidative stress is common in depressed patients (52) and may lead to increased DNA damage along with mitochondrial dysfunction (52). Restoration of these genes may be one of the hallmarks of this herbal medicine. **Table 2** summarizes the functional characterization of 40 recovered IR genes.

**Table 2:** Functional categorization of recovery IR genes.

Functional category	Gene*	IR V-shape	IR reverse V	Description	Ref.
**DNArepair,** **Recombination**	*PMS2*		✓	Elevated levels of mutation in multiple tissues of mice deficient in the DNA mismatch repair gene *Pms2*	(139)
	***REC8***		✓	Meiotic prophase roles of *Rec8* in crossover recombination and chromosome structure	(140)
**Hematopoiesis**	***CDIN1***		✓	The congenital dyserythropoieitic anemias: genetics and pathophysiology	(141)
	***EOGT***		✓	Synergistic regulation of Notch signaling by different O-glycans promotes hematopoiesis	(98)
	*SMARCD2*	✓		A *SMARCD2*-containing m SWI/SNF complex is required for granulopoiesis	(142)
**Cilia**	*AHI1*	✓		*AHI1*, whose human ortholog is mutated in Joubert syndrome, is required for Rab8a localization, ciliogenesis and vesicle trafficking	(61)
	*C21orf58*		✓	Targeting C21orf58 is a Novel Treatment Strategy of Hepatocellular Carcinoma by Disrupting the Formation of JAK2/C21orf58/STAT3 Complex	(159)
	*CELSR2*		✓	CELSR2, Encoding a Planar Cell Polarity Protein, is a Putative Gene in Joubert Syndrome with Cortical Heterotopia, Microophthalmia, and Growth Hormone Deficiency	(62)
	*CEP104*	✓		Joubert Syndrome in French Canadians and Identification of Mutations in *CEP104*	(63)
	***DNHD1***	✓		Bi-allelic variants in *DNHD1* cause flagellar axoneme defects and asthenoteratozoospermia in humans and mice	(143)
	***GBF1***		✓	The Arf GEF *GBF1* and ARF4 synergize with the sensory receptor cargo, rhodopsin, to regulate ciliary membrane trafficking	(105)
	*NAPEPLD*	✓		Small Molecule Activation of NAPE-PLD Enhances Efferocytosis by Macrophages	(83)
	***NPHP1***	✓		Many Genes—One Disease? Genetics of Nephronophthisis (NPHP) and NPHP-Associated Disorders	(70)
**Mitochondria**	*FOXRED1*	✓		Characterization of mitochondrial *FOXRED1* in the assembly of respiratory chain complex I	(75)
	***MFN2***	✓		Mitofusin 2 (*MFN2*) links mitochondrial andendoplasmic reticulum function with insulin signaling and is essential for normal glucose homeostasis	(144)
	***MYH10***		✓	Actin and myosin contribute to mammalian mitochondrial DNA maintenance	(48)
	*NDUFA5*	✓		Supernumerary subunits NDUFA3, *NDUFA5* and NDUFA12 are required for the formation of the extramembrane arm of human mitochondrial complex I	(75)
	***SIGMAR1***	✓		The role of *SIGMAR1* gene mutation and mitochondrial dysfunction in amyotrophic lateral sclerosis	(145)
	***SPG7***	✓		*SPG7* Is an Essential and Conserved Component of the Mitochondrial Permeability Transition Pore	(146)
	*TEFM*		✓	*TEFM* (c17orf42) is necessary for transcription of human mtDNA	(147)
**Inflammation**	***ADCY4***		✓	cAMP metabolism controls caspase-11 inflammasome activation and pyroptosis in sepsis	(117)
	***BRD9***		✓	Bromodomain containing 9 (BRD9) regulates macrophage inflammatory responses by potentiating glucocorticoid receptor activity	(96)
	***CDCA3***		✓	*CDCA3* promotes cell proliferation by activating the NF-kB/cyclin D1 signaling pathway in colorectal cancer	(148)
	*CERT1*		✓	Ceramides as Mediators of Oxidative Stress and Inflammation in Cardiometabolic Disease	(88)
	***CXCL2***		✓	NF-kB and *STAT1* control *CXCL1* and *CXCL2* gene transcription	(43)
	*DDX5*	✓		IL-17D-induced inhibition of *DDX5* expression in keratinocytes amplifies IL-36R-mediated skin inflammation	(149)
	*ERLIN1*	✓		The *ERLIN1-CHUK-CWF19L1* gene cluster influences liver fat deposition and hepatic inflammation in the NHLBI Family Heart Study	(150)
	***FAS***		✓	The Many Roles of *FAS* Receptor Signaling in the Immune System	(151)
	***IL17RB***		✓	Cutting Edge: IL-17B Uses IL-17RA and IL-17RB to Induce Type 2 inflammation from Human Lymphocytes	(152)
	***MYLK***		✓	Myosin Light Chain Kinase: A Potential Target for Treatment of Inflammatory Diseases	(50)
	***NCSTN***	✓		Keratin 5-Cre-driven deletion of *NCSTN* in an acne inversa-like mouse model leads to a markedly increased IL-36a and *SPRR2* expression	(153)
	*NDRG2*	✓		*NDRG2* deficiency exacerbates UVB‑induced skin inflammation and oxidative stress damage.	(110)
	***NFATC4***	✓		*NFAT* is a nerve activity sensor in skeletal muscle and controls activity-dependent myosin switching	(111)
	*NOSIP*	✓		NOSIP, a novel modulator of endothelial nitric oxide synthase activity	(40)
	***OAS2***	✓		*OAS1*, *OAS2*, and *OAS3* Contribute to Epidermal Keratinocyte Proliferation by Regulating Cell Cycle and Augmenting IFN-1- induced Jak1−Signal Transducer and Activator of Transcription 1 Phosphorylation in Psoriasis	(42)
	***PRMT7***	✓		The Role of Protein Arginine Methyltransferases in Inflammatory Responses	(154)
	*ROBO3*	✓		Axon guidance receptor *ROBO3* modulates subtype identity and prognosis via AXL-associated inflammatory network in pancreatic cancer	(92)
	*SLC22A5*	✓		Characterization of exosomal *SLC22A5* (*OCTN2*) carnitine transporter	(155)
	*TRIM16*		✓	*TRIM16* exerts protective function on myocardial ischemia/reperfusion injury through reducing pyroptosis and inflammation via NLRP3 signaling	(156)
	*UBE2T*	✓		Correlations between UBE2T Expression and Immune Infiltration in Different Cancers	(138)
	***USP21***	✓		*USP21* Deubiquitinase Regulates AIM2 Inflammasome Activation	(157)

*Genes detected under the condition of FDR<0.1 are shown in bold. Others were detected at p<0.05.

In short, using IR genes identified after two months of HKT administration, we found significant intron recovery in IR genes, suggesting the efficacy of HKT in depressed subjects. Characterization of 64 recovered protein-coding genes showed a significant impact on inflammation-related genes, consistent with the known anti-inflammatory effects of HKT and highlighting IR as a superior marker for evaluating drug efficacy.

### Pathways for which IR was restored by HKT can be characterized from the IR-DEG interactome

**Figure 7:**
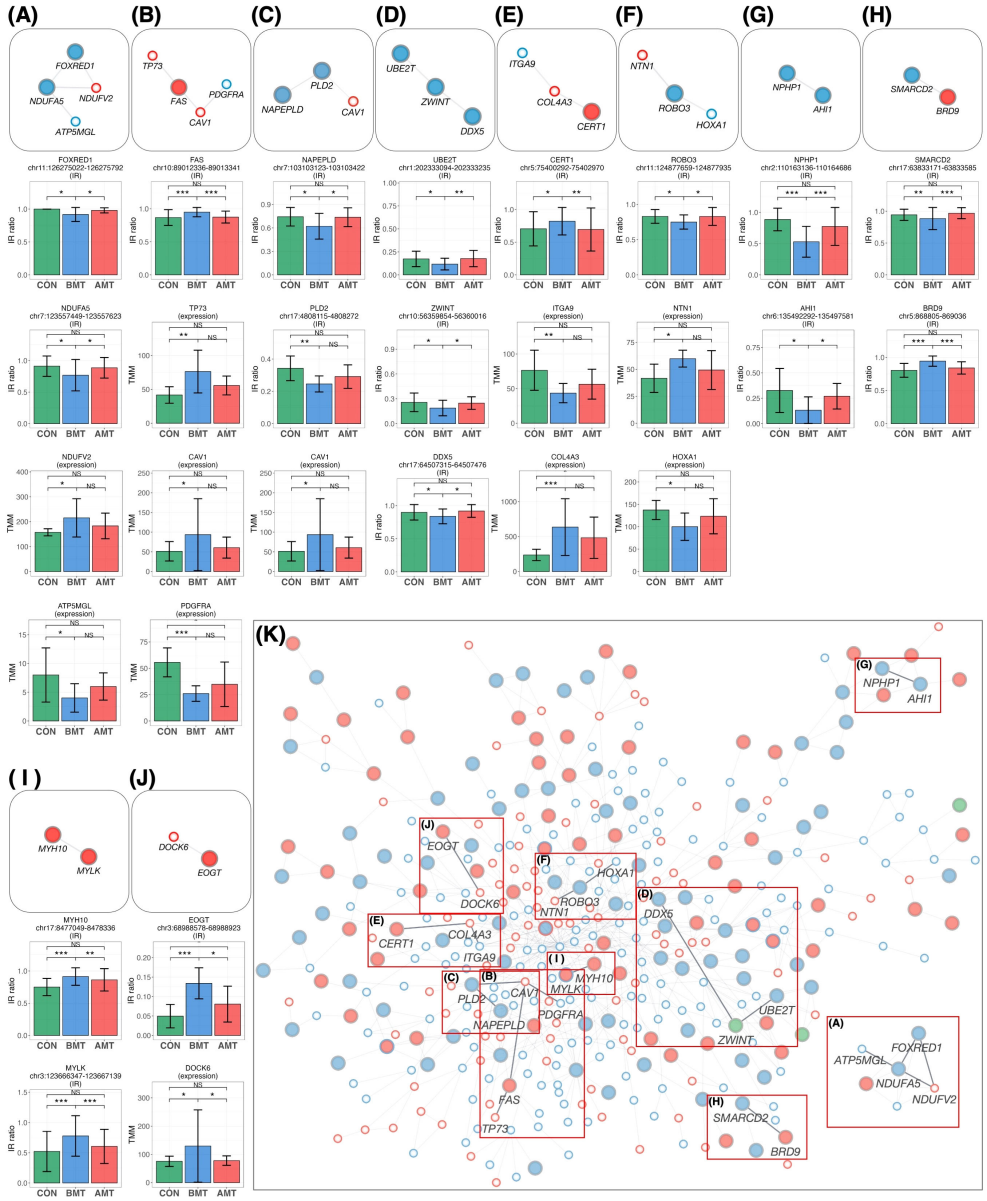
New pathways restored by HKT overlaid on the PPI network. **(A–J)** The network was extracted from the PPI network and then overlaid with the recovered IR and DEG loci. All recovered IR loci except *PLD2* showed a significant difference (*P* < 0.05, FC > 1.2) between BMT and CON and between BMT and AMT. All DEG loci showed a significant difference (*P* < 0.05, FC > 1.2) between BMT and CON, but their significance between BMT and AMT was marginal (*P* < 0.3; likelihood ratio test). Each IR gene is indicated by a large circle, where blue indicates DecIR and red IncIR. DEGs are indicated by a small circle, where blue indicates downregulation and red indicates upregulation. Intron ratio for IR and gene expression levels for DEGs. In the bar graph, asterisks indicate statistically significant differences (*P < 0.05, **P < 0.01, ***P < 0.001, NS: not significant). **(A)** The pathway involving *NDUFA5*, *FOXRED1*, *NDUFV2* and *ATP5MGL* (75) regulates mitochondrial function. **(B)** The *TP73* (76) - *FAS* (77) - *CAV1* (78, 79) - *PDGFRA* (80) pathway is involved in inflammatory signaling. **(C)** The *CAV1* (78, 79) - *PLD2* (81) - *NAPEPLD* (82, 83) signaling pathway is involved in the regulation of lipid metabolism involving caveolae as a vital plasma-membrane sensor. **(D)** The *DDX5* (84, 85) - *ZWINT* (86) - *UBE2T* (87) pathway is involved in the amplification by ubiquitination of an inflammatory signal taken up by *DDX5* via the immune infiltration stimulated by *ZWINT*. **(E)** The *CERT1* (88) - *COL4A3* (89) - *ITGA9* (90) pathway is involved in anti-inflammatory responses. **(F)** The *HOXA1* (91) - *ROBO3* (92) - *NTN1* (93) signaling pathway regulates inflammation. **(G)** See text. **(H)** The *SMARCD2* (94, 95) - *BRD9* (96, 97) signaling pathway mediates inflammatory input activated by *BRD9*, thereby mediating granulopoiesis as an output through activation of *SMARCD2*. **(I)** See text. **(J)** The *EOGT* (98) - *DOCK6* (99) pathway regulates hematopoiesis. **(K)** In the main network, each position of the pathways **(A–H)** is indicated by a red outline.

The 64 protein-coding IR genes (**Figures 6B, C**) and 17 DEGs (**Figure 6E**), both restored by HKT, were overlaid on the IR-DEG interactome (shown in **Figure 5**) to determine whether any of these 81 genes could interact and network with each other. Ten new pathways were found (**Figure 7**). Protein-protein interactions in some of these pathways are known, but to our knowledge this is the first time that these pathways have been shown to be involved in restoring a physiological state. The implications of some of these pathways for the efficacy of HKT are discussed in the later section. Because the IR-DEG interactome was generated independently of HKT treatment, this method offers the possibility that new pathways will be discovered when different drugs are used in similar subjects. Thus, IR-DEG interactome analysis may allow us to uncover new pathways involved in the mechanism of action of different types of drugs, including herbal drugs, and identify their commonalities and unique characteristics.

### Comparison of current data with previous studies

**Figure 8:**
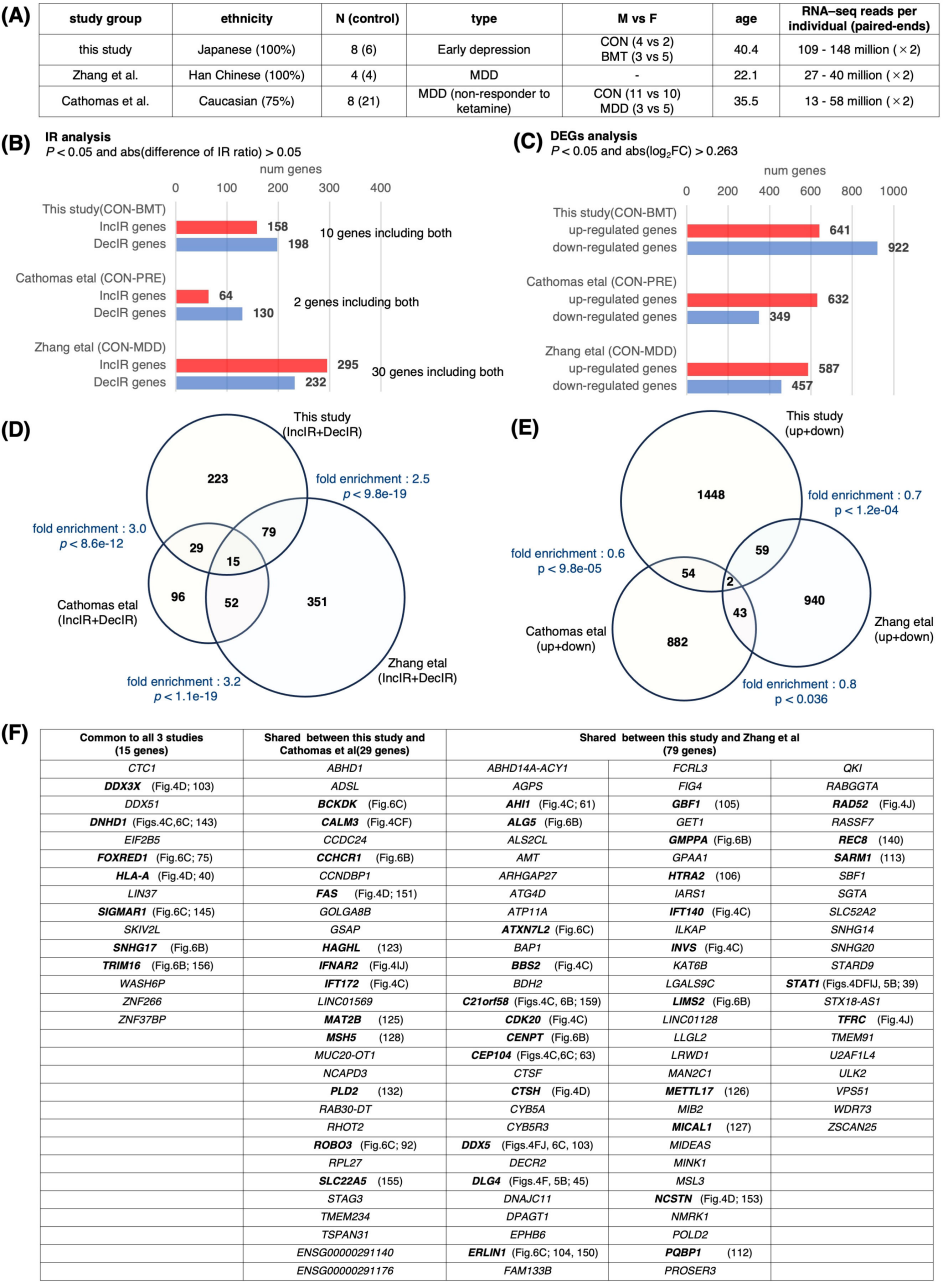
Comparison with previous studies. Transcriptome data from previous depression studies were downloaded from NCBI to obtain IR genes and DEGs. The downloaded data were GSE185855 (Cathomas et al. (158), using CON and PRE nonresponder data) and GSE190518 (Zhang et al. (67), using CON and MDD data) **(A)** A table showing the characteristics of these three studies. **(B)** Bar graph showing the number of genes containing IR in each study. IR genes were defined as those with p < 0.05 and abs(log2FC) > 0.263 using rMATS. **(C)** Bar graph showing the number of DEGs in each study; DEGs were defined as those changed at p < 0.05 and abs (difference of IR ratio) > 0.05 using the same method as in this study. **(D)** Venn diagram of IR genes for each group. The fold enrichment of overlap between each group compared to random was determined, and P values were calculated using Fisher’s exact test. The total number of 4,546 genes from the annotation file (GRCh38 release109) was used for the calculation of fold enrichment. **(E)** Venn diagram showing DEGs for each group. Fold enrichment as in **(D)** and p values were calculated using Fisher’s exact test. The total number of genes was set to 22,000. **(F)** Table with overlapping genes in **(D)**. Genes mentioned in this study are shown in bold.

If there were RNA-seq data on people with symptoms of depression in previous published studies, it would be very useful because it could be compared with our data. However, we thought it would be quite difficult to do such a study. The reason is that most RNA-seq studies of depression to date have been DEG studies, for which 20 million reads per individual is sufficient. However, because IR detection in rMats uses only intron and exon junction sequences, it requires about 100 million reads, which is five times more than DEG studies. Since there are no studies that have read this amount of reads, we thought it was impossible to investigate in this direction. However, we searched again and found two promising studies published by Zhang et al. (67, 2022) and Cathomas et al. (158, 2022). Both studies included patients diagnosed with MDD (**Figure 8A**).

We isolated DEG and IR genes from these two groups (**Figures 8B, C**) and searched for overlapping genes among the three groups, including our study, and made a surprising discovery (**Figures 8D, E**). A large number of overlapping genes were detected among the IR genes (fold enrichment; 2.5~3.2), whereas the DEG genes hardly overlapped among these three groups (fold enrichment; 0.6~0.8). Despite the difference in ethnicity among the three groups, and the fact that our study was not conducted on MDD patients but on subjects with early depression, and that in the case of Cathomas’ study, MDD patients were analyzed from non-responders to ketamine, indicating that the degree of depression was quite heterogeneous (**Figure 8A**), many genes discussed in the previous sections in terms of cilia-related or immunity-related functions (shown in bold in **Figure 8F**) were found to overlap among the three groups or between groups. Overlapping genes detected in these analyses, especially the 15 genes found to be common to these three groups, may be excellent markers of general depression. It is interesting to note that among these 15 genes, 5 genes (*DNHD1, FOXRED1, SIGMAR1, SNHG17*, and *TRIM16*) were restored by HKT administration (**Figures 6B, C**, **8F**), providing the possibility for the general efficacy of this drug on a variety of types of depression including TRD (158; treatment-resistant depression).

When a total of 497 IR genes (IncIR and DecIR combined) isolated from the study of Zhang et al. (67) was compared to the 346 genes detected in our study, 94 genes were common (27.1%; **Figure 8D**). Among these common IR genes, we found cilia-related (*AHI1, BBS2, C21orf58, CDK20, CEP104, DNHD1, GBF1, INVS, PQBP1*) and immune-related (*CTSH, DDX3X, DLG4, HLA-A, STAT1*) genes (**Figure 8D**, **Supplementary Table 5**). Of particular interest is the presence of nine cilia-related genes, *STAT1*, which has the most interactions with immune-related gene sets, and *DLG4*, which has the highest number of links in our analysis of the IR-DEG interactome, all of which we have already discussed in the text as potential markers of depression (See above). We speculate that all the 346 genes represent, to some extent, the stress status of this early presymptomatic stage of depression. The genes that appear naturally in the early stages of depression and those that appear when depression has progressed to MDD are likely to be different. However, the common IR genes found here (27.1%) are the only ones that still play a role when depression has progressed. Incidentally, the overlap between our DEG genes and the genes detected as DEGs in the paper by Zhang et al. (67) was 3.9%. This also proves that the IR gene is a better marker than the DEG gene. It should also be noted that the overlapping genes of Cathomas et al. (158) study with this study and Zhang et al. study (29 + 15 + 52 = 96) are 50% of all IR genes of Cathomas et al. study, which again emphasizes the superiority of IR genes over DEG genes.

In short, re-analyzing previously published RNA-seq data, isolating DEG and IR genes, and comparing them to our data shows that many IR genes are shared. On the other hand, the DEG analysis shows little gene overlap. This suggests that the DEG genes cannot be used as markers as we claimed, but the IR genes can be excellent markers.

## Discussion

### Intron fine-tuning model links IR to protein homeostasis

An important aspect of our study is that the data show that IR can be used as an alternative clinical tool for the diagnosis of depression instead of traditional DEG-based methods. Roughly speaking, GO terms enriched in IncIR genes corresponded to GO terms of upregulated DEGs, whereas GO terms enriched in DecIR genes corresponded to GO terms of downregulated DEGs. Recently, we presented an IR fine-tuning model in which an increase in IR leads to a decrease in the amount of mature cytoplasmic mRNA (and thus a decrease in cytoplasmic protein), whereas a decrease in IR leads to an increase in mature cytoplasmic mRNA (and thus an increase in cytoplasmic protein) (29). Taken together, this suggests that the IR fine-tuning mechanism is at work in regulating intracellular protein homeostasis, i.e., when protein levels are physiologically high, introns in the corresponding gene increase, followed by a decrease in mRNA expression, and conversely, when protein levels are low, IR decreases and mRNA increases. In other words, IR is thought to be a molecular mechanism that detects changes in the amount of proteins required in the cytoplasm due to physiological adaptation in response to stress and optimizes the amount of cytoplasmic proteins.

### The sensor role of IR is evolutionarily conserved

As can be easily imagined from the model described above, genes that undergo IR have a sensor role. Indeed, in the literature describing the gene functions of many of the depression-related IR genes we identified, the article titles often include the word ‘sensor’ or ‘regulate’ (**Table 1**). Therefore, if an IR is observed in depressed patients for a gene whose sensor role is not reported in the literature, analysis of the IR gene may reveal a new, previously unidentified, regulatory sensor role for the gene in clinically depressed patients.

In many cases, the IR genes we identified did not correspond to genes in the DEGs themselves. Of the 63 DEGs detected at FDR<0.1 (**Supplementary Figure 4**), none is actually subject to IR. DEGs are often reflect quantitative aspects, whereas IR genes are more qualitative. By analogy, the DEG is the soldier, the manual worker, whereas the IR genes are the commander in chief. A typical example of the qualitative difference between DEGs and IR genes is secreted proteins, which are sometimes detected as DEGs (indeed, half of the upregulated genes we identified were immunoglobulins; **Supplementary Figure 2**) but not as IR genes. This is because these proteins are secreted from cells via the Golgi and are therefore not captured by the homeostasis detection mechanism in the cytoplasm (29).

Based on our previous publications (27–29), it is likely that some genes (about 10~20%) of the 20,000-30,000 genes in the mammalian genome were originally ASSIGNED to play a sensor role by retaining introns during stress (see also Introduction). In budding yeast, for example, introns may play a mediator role in monitoring the physiological state of the cell (53, 54). Thus, the sensor function of IR genes is likely to be evolutionarily conserved, as described in the present study. However, the molecular mechanisms by which IR genes sense the physiological stresses experienced by cells and contribute to homeostasis (29) remain to be elucidated.

### IR is an excellent marker for diagnosing depressive states and is superior to DEG

Many researchers have analyzed DEGs between cases and controls to identify markers of depression. It has been found that even when the top 10 genes with the highest expression variability are examined, the data differ from experiment to experiment and are not consistent (55). As our current study shows (**Figure 8**), IR variation is likely to be a more sensitive marker than DEGs for the diagnosis of depression. Consider the following practical interpretation: a 10% variation in immunoglobulin levels detected by DEG does not necessarily represent the physiological state of a particular individual, but a 10% decrease in the IR of the inflammation sensor STAT1 (39) could result in a 10% increase in the amount of cytosolic STAT1 protein, thus having a significant impact on immune homeostasis. The accumulation of various studies to date suggests that the cause of depression is polygenic as an explanation for the fact that DEG data fluctuate and do not agree from experiment to experiment (56), but the failure to find a better marker gene in the DEG is not so much that depression is polygenic, likely due to the nature of the DEGs themselves.

### Examples of markers for the diagnosis of depressive states

Among the IR genes we identified, the 34 genes identified as immune-related with p<0.05, of which 17 genes were detected under the condition of FDR<0.1 (**Figure 4D**), are likely to be good candidates for excellent markers of depression. In addition, the expression of immune-related genes changes in response to depression, and the IR genes most likely to interact with them would likely change with depression. Therefore, IR genes with a high ranking for interaction with immune-related genes, as shown in **Figures 4F, I, J** also have great potential as markers for depression. Hub IR genes with many connections in the interactome (**Figure 5B**), which we discussed earlier, are also good candidates.

Regarding common markers of depression, a particular highlight of the present study is the detection of a number of cilia-related genes (35 genes with p<0.05 or 19 genes with FDR<0.1; **Figure 4C**) as IR genes. This may reflect the functional stress state of cilia as antennae in leukocyte cells, although the presence of cilia on leukocyte cells is controversial (57). It is known that when dendritic cells present antigens to T cells, these two cell types form structures known as immunological synapses, in which the internal structure of the T cell resembles that of cilia (58–60). Therefore, the observation of IR in ciliary genes may represent a failure of T cells to recognize antigens during depression. This interpretation is consistent with the observations that several T cell activation genes were downregulated (**Figure 1E**) and that those involved in the T cell signaling pathway were characterized as DecIR genes (**Figure 3D**).

Of the 1336 cilia genes currently known, IR was observed in 35 genes with p<0.05 (19 genes with FDR<0.1) in this study (**Figure 4C**). Surprisingly, of the 37 cilia-specific genes currently known to cause Joubert syndrome, six (*AHI1* (61), *CELSR2* (62), *CEP104* (63), *IFT172* (64), *NPHP1* (65), *TMEM107* (66)) were among the IR genes. In addition, IR was restored for four of these six genes in response to treatment with HKT (**Figures 6B, C**). Thus, the Joubert syndrome genes appear to be frequently IR and highly responsive to HKT, suggesting that these genes are excellent candidates for marker genes for depression. Indeed, *AHI1* and *CEP104* were found to overlap between the IR genes of the present study and those of Zhang et al. (67), and *IFT172* was found to overlap between those of the present study and those of Cathomas et al. (158) (**Figure 8**). *AHI1* and *NPHP1* were also found to be involved in pathways for recovery as described in the next section (see **Figures 6C**, **7G**). There are different types of depression, ranging from mild to severe depression, and some respond to medication, while others do not. Stratifying the correspondence between depression and the IR gene is one of the important issues for future clinical research.

### Ten characteristic pathways represented by efficacies of HKT

Mapping of HKT-responsive IR genes and DEGs onto the IR-DEG interactome revealed 10 pathways for which the IR of certain genes was recovered in concert with that of other genes. Although each of these 10 pathways deserves detailed investigation (most of them were newly discovered in this study), it is important to emphasize that, in each of these 10 pathways, changes in the IR of one gene were linked to changes in IRs or DEGs, forming a single functional unit (which we call a gear). We will discuss a few of these. The first is the *NPHP1*-*AHI1* pathway (**Figure 7G**). As mentioned above, these two genes are involved in cilia function, and mutations cause a ciliopathy called Joubert syndrome (61, 68–70). Yeast two-hybrid analysis revealed that jouberin (encoded by *AHI1*) can interact with nephrocystin (encoded by *NPHP1*) (71). The two proteins form a heterodimer, and a mutation in *AHI1* (V443D) that prevents heterodimer formation alters the intracellular localization of AHI1 and NHPH1 so that the two proteins can, although not always, behave as if they were one protein (61). It is interesting to note that the IR of these two mRNAs is reduced in depression and that the IR of both mRNAs was restored to that of the healthy state in response to treatment with HKT (**Figure 7G**). In other words, the mRNAs transcribed from these two genes seem to be under the same control mechanism of RNA processing, as if they were the same mRNA. These observations remind us of a model we recently proposed, i.e., that there may be a novel mechanism that senses the correct level of functional proteins in the cytoplasm and transmits this information to the nucleus to regulate the level of IR (29). If such a mechanism exists, it would imply that IR of *AHI1* and *NPHP1* is regulated by a common factor.

In the case of the *MYLK*-*MYH10* pathway (**Figure 7I**), inflammatory inputs activate MYLK and phosphorylate the L-chain of myosin (50). This causes the contracted L-chain of myosin to transmit information to the H-chain, which in turn regulates the copy number of mitochondrial DNA, which is tightly bound to the non-muscle H-chain (48). In this biological GEAR, the input is inflammation and the output is the control of mitochondrial DNA copy number. The GEAR functions in these ten pathways, including the two already postulated, remain to be demonstrated biochemically, but brief outlines of the hypothetical pathways are given in the legend of **Figure 7**.

**Figure 7K** shows the mapping of the 10 pathways for which IR was restored by HKT on the IR-DEG interactome described in this study. This interactome was generated using data from depressed patients and controls only. Thus, if a drug other than HKT (i.e., with a different action) was used, new pathways restored by the drug could be detected. Accordingly, this IR-DEG interactome should be useful for assessing the efficacy of individual drugs, including herbal medicines, and for identifying new pathways affected by drugs.

In conclusion, our results show that IR can be an excellent marker for depression. The combination of network analysis and analysis of drug-responsive IR genes may also reveal novel drug action pathways. The strategy presented here is not limited to the analysis of depression, but could be applied to any disease.

## Data Availability

The original RNA-seq datasets have been deposited in the DDBJ Sequence Read Archive under accession numbers DRR540207–DRR540228, which are linked to the BioProject accession number PRJDB17815.
