# Supplementary material for: Intron retention as an excellent marker for diagnosing depression and for discovering new potential pathways for drug intervention
Source: Front Psychiatry. 2024 Sep 19;15:1450708. doi: 10.3389/fpsyt.2024.1450708 (PMC11446786; doi:10.3389/fpsyt.2024.1450708)
Supplement: Supplementary file 1 [file DataSheet1.pdf]

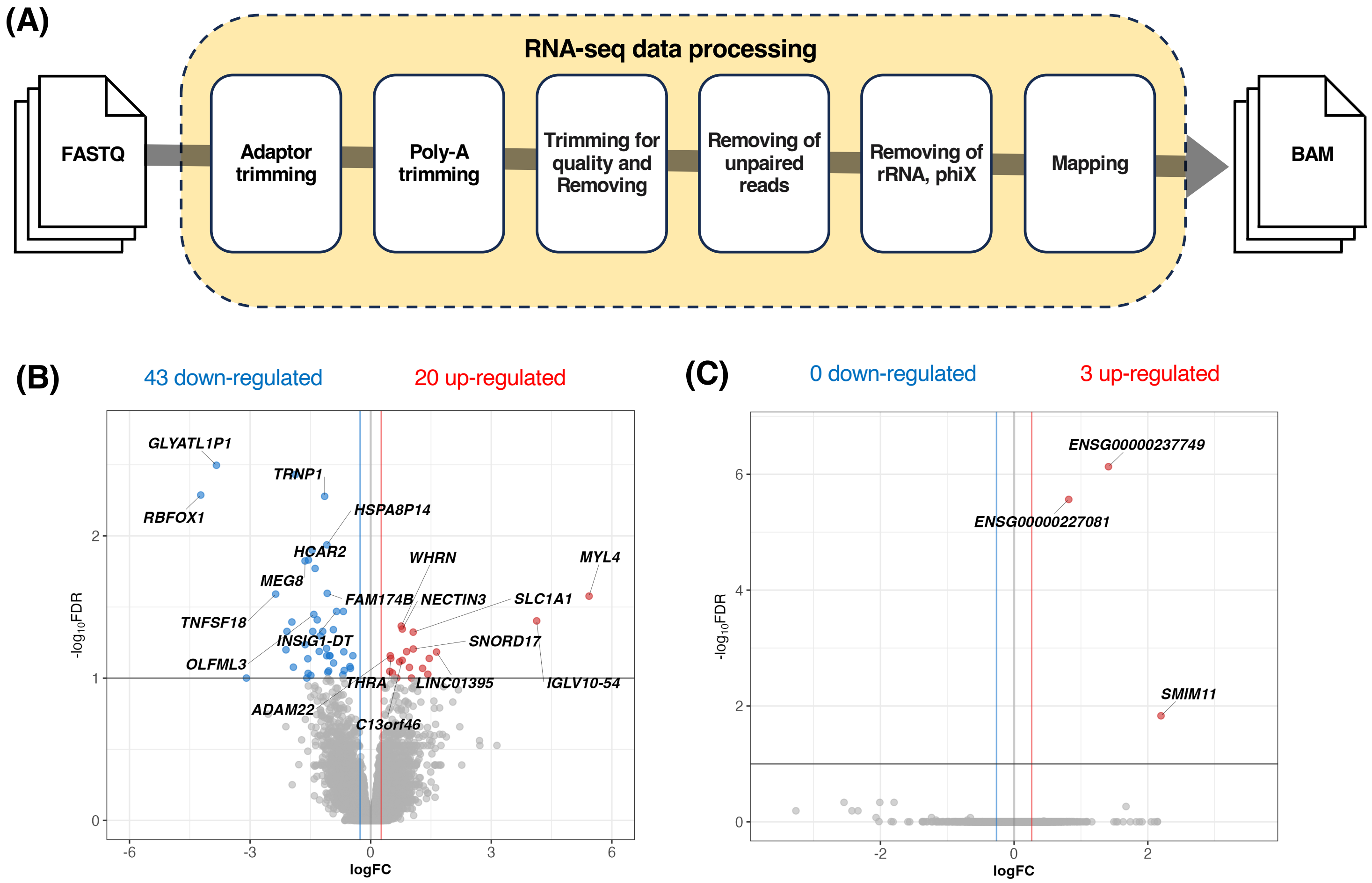

Supplementary Figure 1. Altered RNA expression in depressed patients. (A) Processing flow of RNA-seq data. (i) Adaptor trimming using by cutadapt. (ii) Poly-A trimming using by fastx tool kit. (iii) Trimming for quality and removing using by fastx tool kit. (iv) Removing of unpaired reads using by Trimmomatic. (v) Removing of rRNA, phiX using bu tophat2 and samtools. (vi) Mapping using by Hisat2. See Methods section for details. (B) Volcano plot showing differential expression changes between CON and BMT. Red indicates logFC > 0.263 (FC > 1.2) and false discovery rate (FDR) < 0.1. Blue indicates logFC > -0.263 (FC < 1/1.2) and FDR < 0.1. (C) Volcano plot showing differential expression changes between BMT and AMT.
