# Supplementary material for: Intron retention as an excellent marker for diagnosing depression and for discovering new potential pathways for drug intervention
Source: Front Psychiatry. 2024 Sep 19;15:1450708. doi: 10.3389/fpsyt.2024.1450708 (PMC11446786; doi:10.3389/fpsyt.2024.1450708)
Supplement: Supplementary file 2 [file DataSheet2.pdf]

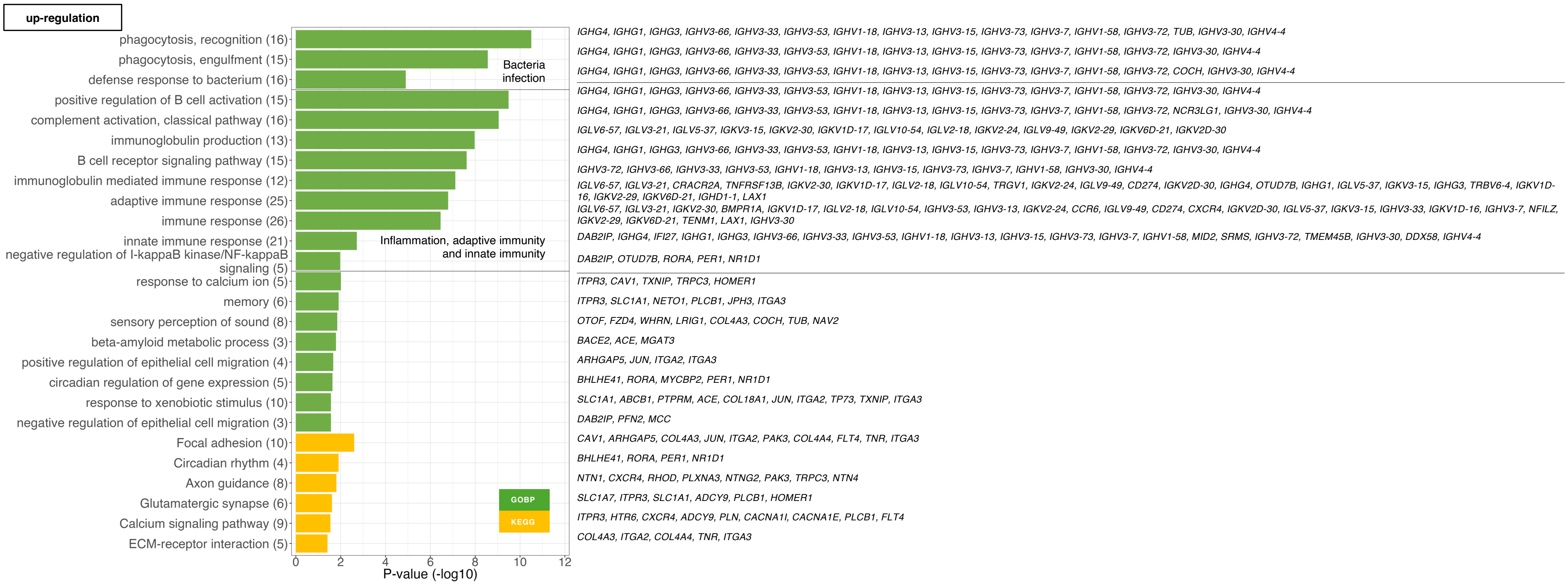

Supplementary Figure 2. Bar chart of enrichment analysis of biological process gene ontology and KEGG pathway terms using up-regulated genes in BMT.
