# Supplementary material for: Intron retention as an excellent marker for diagnosing depression and for discovering new potential pathways for drug intervention
Source: Front Psychiatry. 2024 Sep 19;15:1450708. doi: 10.3389/fpsyt.2024.1450708 (PMC11446786; doi:10.3389/fpsyt.2024.1450708)
Supplement: Supplementary file 3 [file DataSheet3.pdf]

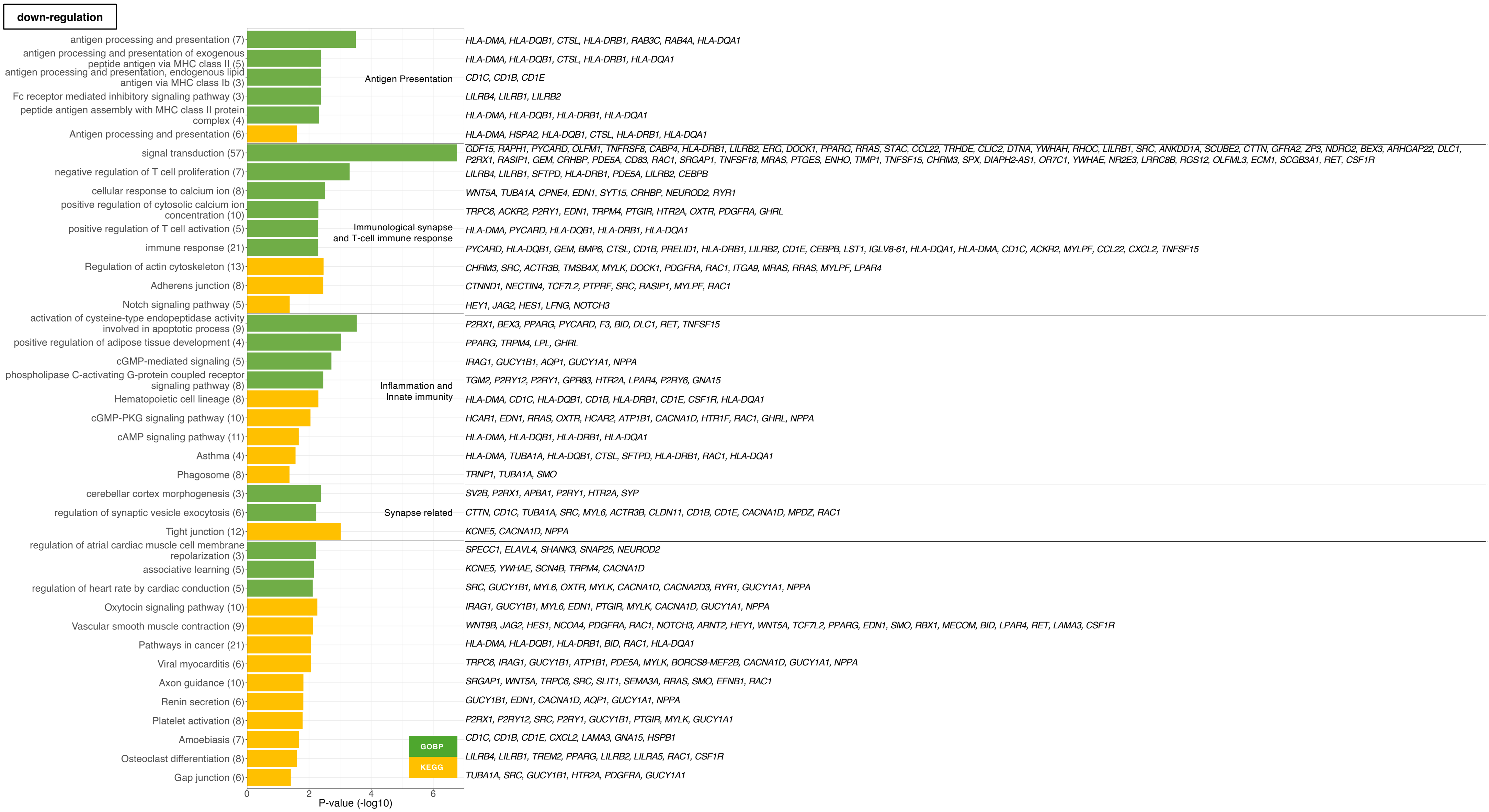

Supplementary Figure 3. Bar chart of enrichment analysis of biological process gene ontology and KEGG pathway terms using down-regulated genes in BMT.
