# Supplementary material for: Intron retention as an excellent marker for diagnosing depression and for discovering new potential pathways for drug intervention
Source: Front Psychiatry. 2024 Sep 19;15:1450708. doi: 10.3389/fpsyt.2024.1450708 (PMC11446786; doi:10.3389/fpsyt.2024.1450708)
Supplement: Supplementary file 4 [file DataSheet4.pdf]

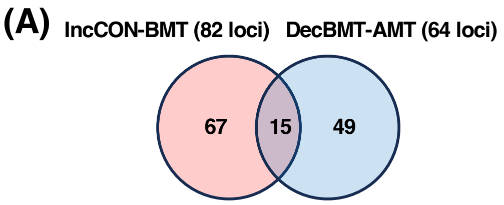

**reverse V-shape**<br><br>15 loci,<br>15 genes,<br>15 protein-coding	***ADCY4, BRD9, C21orf58, CCHCR1, CDCA3, CENPT, CXCL2, FAS, GMPPA, MYLK, POLR3A, REC8, RGP1, SKIC3 (TTC37), TVP23C***

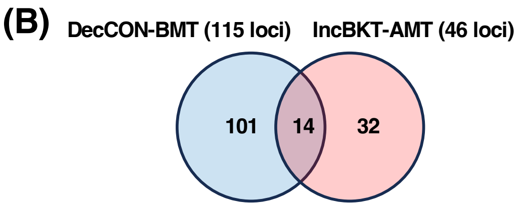

**V-shape**<br><br>14 loci,<br>13 genes,<br>11 protein-coding	***BCKDK, DNHD1, FAM131B, KMT5B,*** *LINC01569*, ***NCSTN, NFATC4, NPHP1, OAS2, PCSK4, SMC4, SPG7,*** *ZNF37BP*

Supplementary Figure 4. Recovery IR loci for cases using a threshold of FDR <0.1. (A) Venn diagram of reverse V-shape comparing 82 loci with significantly increased IR between CON and BMT and 64 loci with significantly decreased IR between BMT and AKT. (B) Venn diagram of V-shape comparing 115 loci with significantly decreased IR between CON and BMT and 46 loci with significantly increased IR between BMT and AKT. The threshold for significance of the increase or decrease in IR ratio was set at FDR < 0.1.
