# Supplementary material for: Intron retention as an excellent marker for diagnosing depression and for discovering new potential pathways for drug intervention
Source: Front Psychiatry. 2024 Sep 19;15:1450708. doi: 10.3389/fpsyt.2024.1450708 (PMC11446786; doi:10.3389/fpsyt.2024.1450708)
Supplement: Supplementary file 5 [file DataSheet5.pdf]

**(A)**

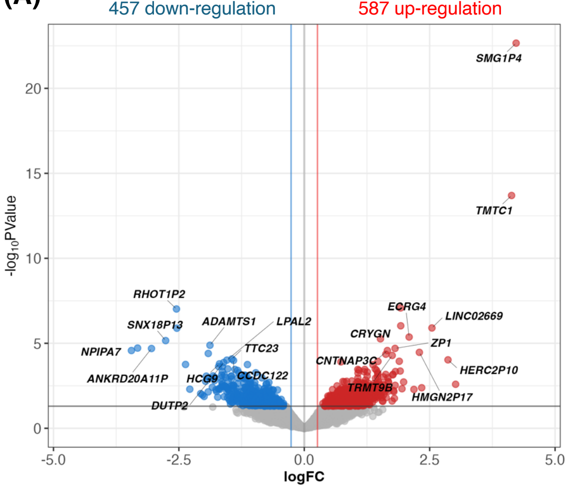


**(B)** **DEGs**

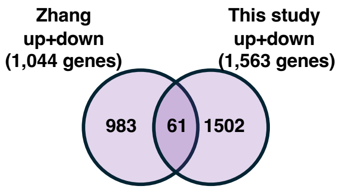


**shared 61 genes**

| | | | |
|---|---|---|---|
| *BATF3* | *HES1* | *PTPRN2-AS1* | *UBE2SP1* |
| *C1DP1* | *HEY1* | *RASIP1* | ENSG00000227678 |
| *CATIP* | *IGHGP* | *RASL11A* | ENSG00000228463 |
| *CCDC38* | *IGLV6-57* | *RBMS3* | ENSG00000229425 |
| *CD81-AS1* | *INSIG1-DT* | *RN7SL388P* | ENSG00000236905 |
| *CDKN1C* | *IRAG1* | *SLC27A2* | ENSG00000237101 |
| *CHD5* | *KIR2DL4* | *SMG1P2* | ENSG00000269534 |
| *CRHR2* | *LCN2* | *SMIM11* | ENSG00000270802 |
| *CTDSPL2-DT* | *LILRA5* | *SNRPGP4* | ENSG00000272088 |
| *DEPTOR* | *LINC02371* | *SNX2P1* | ENSG00000278236 |
| *DUSP5-DT* | *LPAR4* | *TCF7L2* | ENSG00000278655 |
| *ECM1* | *NAIPP2* | *TMEM158* | ENSG00000280474 |
| *EPS8* | *OLFM1* | *TMEM45B* | ENSG00000286285 |
| *FA2H* | *PAK3* | *TMSB4XP1* | |
| *GFRA2* | *PLA2G10* | *TPBG* | |
| *GUSBP16* | *PTPRM* | *TRIM10* | |

**(C)** **Valiable IR loci**

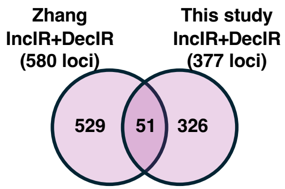


**shared 51 loci**

| | |
|---|---|
| AGPS,chr2:177442236-177442405 | LIMS2,chr2:127640319-127640894 |
| AHI1,chr6:135492292-135497581 | LINC01128,chr1:852111-852669 |
| ALG5,chr13:36993673-36995423 | LLGL2,chr17:75574261-75574608 |
| ALS2CL,chr3:46675977-46676243 | LRWD1,chr7:102465628-102465815 |
| ARHGAP27,chr17:45404097-45404443 | MAN2C1,chr15:75359154-75359326 |
| ATP11A,chr13:112857921-112859391 | MIDEAS,chr14:73719009-73719303 |
| ATXN7L2,chr1:109489101-109489928 | NMRK1,chr9:75069103-75069893 |
| ATXN7L2,chr1:109491718-109492025 | POLD2,chr7:44117248-44117582 |
| C21orf58,chr21:46301714-46301995 | PROSER3,chr19:35766956-35767070 |
| CTC1,chr17:8228630-8229300 | QKI,chr6:163563720-163564665 |
| CYB5A,chr18:74255776-74260913 | RASSF7,chr11:563318-563394 |
| CYB5R3,chr22:42636599-42636713 | REC8,chr14:24173042-24173124 |
| DDX3X,chrX:41343352-41344028 | SARM1,chr17:28388277-28388358 |
| DLG4,chr17:7194001-7194317 | SLC52A2,chr8:144359066-144359186 |
| DNHD1,chr11:6548041-6548208 | SNHG20,chr17:77088950-77089151 |
| ERLIN1,chr10:100185638-100185902 | STARD9,chr15:42663491-42663818 |
| FAM133B,chr7:92577677-92578148 | STAT1,chr2:190979002-190980618 |
| FCRL3,chr1:157697425-157697657 | STX18-AS1,chr4:4649110-4649368 |
| FIG4,chr6:109788957-109789592 | TMEM91,chr19:41382922-41383559 |
| GBF1,chr10:102362629-102363254 | TRIM16,chr17:15683158-15684194 |
| GET1,chr21:39406059-39406560 | ULK2,chr17:19771068-19776307 |
| HLA-A,chr6:29943027-29943266 | VPS51,chr11:65107373-65107579 |
| HTRA2,chr2:74531412-74531595 | WASH6P,chrX:156022460-156022697 |
| ILKAP,chr2:238194332-238194780 | ZNF266,chr19:9418896-9419049 |
| INVS,chr9:100292437-100292945 | ZSCAN25,chr7:99619998-99621371 |
| LGALS9C,chr17:18492546-18492695 | |

**(D)**

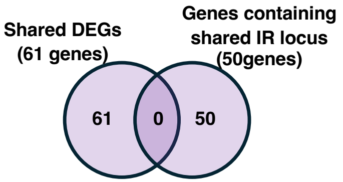


**Supplementary Figure 5. Comparison of DEG/IR between the MDD data of Zhang *et al* and this study.**

(A) Volcano plots using transcriptome data between CON and MDD nadfrom the MDD data of Zhang et al. The horizontal axis shows the $\log_2$ fold-change of BMT/CON, and the vertical axis shows $-\log_{10}$ *P*-values. Red dots denote significantly upregulated genes (FC (fold change) > 1.2 and $P < 0.05$), blue dots denote significantly downregulated genes (FC < 1/1.2 and $P < 0.05$), and grey dots indicate no significant difference in expression (likelihood ratio test). (B) Comparison of RNA expression between the MDD data of Zhang *et al* and this study. Venndaigram of comparison using up-regulated + down-regulated genes. (C) Comparison of variable intron retention between the MDD data of Zhang *et al* and this study. Venndaigram of comparison using IncIR + DecIR loci. (D) . Venndaigram between shared DEGs (Fig.S5B) and Genes containing shared IR locus (Fig. S5C).
